# Patterns of working hour characteristics and risk of sickness absence among shift-working hospital employees: a data-mining cohort study

**DOI:** 10.5271/sjweh.3957

**Published:** 2021-06-29

**Authors:** Tom Rosenström, Mikko Härmä, Mika Kivimäki, Jenni Ervasti, Marianna Virtanen, Tarja Hakola, Aki Koskinen, Annina Ropponen

**Affiliations:** Finnish Institute of Occupational Health, Helsinki, Finland; Department of Psychology and Logopedics, Faculty of Medicine, University of Helsinki, Helsinki, Finland; Clinicum, Faculty of Medicine, University of Helsinki, Helsinki, Finland; Department of Epidemiology and Public Health, University College London, London, United Kingdom; School of Educational Sciences and Psychology, University of Eastern Finland, Joensuu, Finland; Division of Insurance Medicine, Department of Clinical Neuroscience, Karolinska Institutet, Stockholm, Sweden

**Keywords:** employee scheduling, nurse rostering, occupational health, permutation distribution clustering, sick leave, shift work, shift worker

## Abstract

**Objectives:**

Data mining can complement traditional hypothesis-based approaches in characterizing unhealthy work exposures. We used it to derive a hypothesis-free characterization of working hour patterns in shift work and their associations with sickness absence (SA).

**Methods:**

In this prospective cohort study, complete payroll-based work hours and SA dates were extracted from a shift-scheduling register from 2008 to 2019 on 6029 employees from a hospital district in Southwestern Finland. We applied permutation distribution clustering to time series of successive shift lengths, between-shift rest periods, and shift starting times to identify clusters of similar working hour patterns over time. We examined associations of clusters spanning on average 23 months with SA during the following 23 months.

**Results:**

We identified eight distinct working hour patterns in shift work: (i) regular morning (M)/evening (E) work, weekends off; (ii) irregular M work; (iii) irregular M/E/night (N) work; (iv) regular M work, weekends off; (v) irregular, interrupted M/E/N work; (vi) variable M work, weekends off; (vii) quickly rotating M/E work, non-standard weeks; and (viii) slowly rotating M/E work, non-standard weeks. The associations of these eight working-hour clusters with risk of future SA varied. The cluster of irregular, interrupted M/E/N work was the strongest predictor of increased SA (days per year) with an incidence rate ratio of 1.77 (95% confidence interval 1.74–1.80) compared to regular M/E work, weekends off.

**Conclusions:**

This data-mining suggests that hypothesis-free approaches can contribute to scientific understanding of healthy working hour characteristics and complement traditional hypothesis-driven approaches.

Shift work prevalence is 22% in the European working age population (1) and 20–25% in developed countries (2). Shift work has been linked to increased risk of sickness absence (SA), occupational injuries, depression and various chronic conditions including the metabolic syndrome, type 2 diabetes mellitus, and coronary heart disease (2–6). However, shift work captures a wide range of working hour arrangements, such as fixed night shift work; rotating eight-hour shifts (bulk shift at a.m., p.m., and night); and irregular shift work characterized by a non-standard schedule with varying start and finish times, shift lengths, and rest periods between shifts (7). Working hour characteristics in shift work can also vary in terms of the length of the working hours (eg, the length of work shifts or work shift spells), shift intensity (defined by time between the individual shifts), and time of the day (timing of work shifts) (8). It remains uncertain which specific patterns of shift work are harmful as the concept of shift work captures a wide and heterogeneous set of working hour arrangements

To date, most shift work research has been focused on one or few pre-defined shift work patterns rather than the full range of different shift work patterns. This approach does not capture interrelations between working hour characteristics, which can have different effects on health, and it may also fail to capture relevant temporal aspects of the target working hour characteristic. Wherein traditional statistical approaches often operationalize and test one confounding variable at a time, data-mining approaches can take in large quantities of data and automatically find patterns of confounding. Long work shifts, for example, are more common in hospitals during the nights and are often followed by longer time-off (9, 10). By implication, quick returns (ie, short recovery after shift) are inversely associated with long shifts, which may contribute to the unexpected findings in studies that are focused on a single working hour characteristic only because both quick returns (11) and long working hours (3, 4) are associated with increased risk for negative health and well-being effects. An earlier study found that long work shifts were associated with *less* sick leaves among hospital employees working irregular shifts raising speculations that the risk estimates for long shifts were confounded by other protective effects, such as longer recovery periods after the long shifts (12). More generally, the researcher- or hypothesis-derived pre-defined shift work patterns represent only a small subset of possible patterns, whereas modern data-mining tools would allow systematic exploration through a vastly larger space of possible patterns present in the given data.

In this study, we used data-mining tools to define working hour patterns in shift work over prolonged periods based on the following shift-specific parameters: work shift length, between-shift rest period, and shift starting time. The main aims of the paper were to: (i) characterize working hour patterns in shift work by means of permutation distribution clustering as a data-mining tool, and (ii) study associations between these shift work patterns and sickness absence.

## Methods

### Study population

We used working hour data from employees of the Hospital District of Southwest Finland. A total of 6029 hospital employees (506 men and 5523 women, mean age 39.37 years, range 18–70 years) were included in the analyses. Their work contract, or succession of contracts under the same employer, had lasted ≥3 years (3×365 days) basically uninterrupted (pauses of ≤4 days were allowed). The longest uninterrupted sequence of data per employee was used when more than one ≥3-year sequences were available. The included employees were also working full-time in all 1-year time windows within their longest uninterrupted data sequence. Full-time work was defined as ≥150×7.75 realized work hours per year (ie, in all 52-week windows; only ~8% did not satisfy the condition). We excluded physicians and, based on the work contracts, the employees having an office-hour contract so we could concentrate on hospital employees in shift work (mostly registered nurses by occupation).

### Data on working hour characteristics

The records of working hours were drawn from Titania^®^ shift-scheduling program including the final realized working hours used for payroll. For each employee, we derived work shift lengths, between-shift rest periods (time after index shift and before the next shift), and shift starting times for all available successive shifts to capture the entirety of the employees’ recorded work time from 1 January 2008 to 27 August 2019 in a format conducive of time-series clustering. Therefore, the data contained the three work shift-related dimensions arranged along the dimension of work-shift succession (technically, a discrete 3-dimensional time series), and could contain up to 7845 scalar-valued observation values per employee. Work shifts starting immediately after a previous one (with 1-minute precision) were considered to be part of the previous work shift (ie, they were removed after adding their hours to the previous shift). Unreliable short entries (all <3 hours) were removed according to previously defined procedures (8).

### Shift-ergonomics risk score

We also evaluated the data-driven clusters against risk scores derived from the Finnish Institute of Occupational Health (FIOH) recommendations for shift ergonomics (13). To evaluate the novel content in our cluster solution, it was preferable to compare them to the existing constructs for shift ergonomics. The FIOH recommendations have been integrated into the data-generating Titania shift-scheduling software and are used for the automatic evaluation of work shift patterns (8, 13, 14). We used three main characteristics currently considered most important by FIOH (8, 13) to derive work burden (overload) risk scores (range 0–3). The risk scores were based on (i) total work time between two days off work, (ii) number of night shifts over a 3-week period, and (iii) number of quick returns between two days off work (see supplementary material, appendix A for quantitative definitions https://www.sjweh.fi/article/3957).

### Sickness absence data

The data on SA were derived from working hour records, which include dated indicators for absence due to sickness but no diagnosis (15, 16). SA was selected as an objective register-based outcome of high interest but was not used for deriving working hour characteristics clusters.

### Statistical analysis

*Time series clustering*. Our first aim was to detect clusters of different patterns amongst the 3-dimensional working hour characteristics time series ${\left(\left[x_{t,w},x_{t,r},x_{t,s}\right]\right)}_{t=1}^{T}$ where *t* index successive work shifts, *w* working hours, *r* rest-hours (recovery) after the work shift, and *s* work shift start time in hours $x_{t,s}\in [0,24)$ o’clock; note, night shifts get implicitly represented by work shift start time and length). That is, we conducted time series clustering for data where each 3-dimensional time series represented a unique employee. We aimed at a maximally data-driven (hypothesis-free and non-parametric) approach, sensitive to patterns rather than mean levels, and robust to variation in mean levels and extreme values (for generalizability and reproducibility). Amongst the many alternatives (21, 22), we considered permutation distribution clustering (PDC) method as the most fitting starting point (23, 24).

PDC is an approach to cluster a set of time series to subsets of time series that show high within-subset ‘similarities’ and lower between-subset similarities. The PDC method is defined by the distance metric it gives to two distinct time series to quantify the similarity of their probability distributions. To arrive at the most suitable number of subsets, or clusters, we used the hierarchical agglomeration with complete linkage for clustering and Bayesian information criterion (BIC) to evaluate model fit per number of clusters (23, 24).

We used the default distance function of the pdc R package version 1.0.3, the “symmetric alpha divergence”, which represents distance between two discrete probability distributions (23, 24). To map time series onto probability distributions, the PDC method embeds a time series $X=\{x_{i}{\}}_{i=0}^{T}$ sampled at equal intervals *i* onto a *t*-delayed *m*-embedding

$X'={\left(\left[x_{i},x_{i+t},x_{i+2t},x_{i+\left(m-1\right)t}\right]\right)}_{i=0}^{T'}$

with a total of T'=T−(m−1)t elements. Here, *m* = 7 and *t* = 1 were selected by an entropy heuristic (23, 24). Each element of the embedding is thus a set with *m* elements (ie, *m* successive original time-series values) that can be sorted to ascending order by an appropriate permutation function (for instance, a function π: {2,3,1} ↦ {1,2,3}, if *m* = 3). Several elements of the embedding may get sorted by the same permutation (eg, the replacement {2,3,1} ↦ {1,2,3}), but many permutations are needed to sort them all and their rates depend on the original time series. An empirical permutation distribution for a time series is constructed by sorting each member of its embedding and by counting frequencies for the ensuing permutations, or *codewords*, $\pi \in S_{m}$ , where *S_m_* is the set of all *m*-permutations (ie, size of the set is $\left|S_{m}\right|=m!$ and we get T' samples on it per univariate time series). These permutation distributions are called *codebooks* and each employee has three of them, one for each dimension of their working hour characteristic time series. The total distance between two employees is a square root of the sum of their squared symmetric alpha divergences (technical note: this “divergence” also happens to be a “distance”). After the clusters were derived, their contents were explored for interpretation by plotting specific examples and densities of probability distributions with the ks R package, version 1.11.7 (default kernel).

*Poisson regression for predicting sickness absence*. We used Pearson’s chi-squared and Welch’s t-tests for simple two-group comparisons, whereas Poisson regression models were used to study associations between time-series clusters and SA days, with the unit days per year (ie, per 365 days). We modeled a count outcome with offset log(*T*/365), where *T* denotes number of days the employee was followed. Regression coefficients are reported as incidence rate ratios (IRR) and their 95% confidence intervals (CI). To improve comparability between binary and continuous regression inputs, we standardized continuous inputs by subtracting mean and dividing by 2 standard deviations (26).

We first regressed SA on independent variables over the entire follow up periods (cross-sectionally) and then (prospectively) regressed SA from latter halves of the follow up periods on independent variables defined from the first halves of the periods. That is, we defined the first half of an employee-specific time series as the past and the second half as the future. The FIOH risk scores were recomputed for the ‘past’ only to support prospective regression modeling. The cluster memberships were also recomputed using only the first half of the employee-specific time-series data.

To assign cluster membership based on half the original time series, we recomputed the codebooks from the selected data half and then investigated their distances from all employees’ codebooks based on all of their data. The assigned ‘past’ cluster membership was that of the nearest neighbor in original full-data codebooks. Cluster assignments were the same as for the full data for 99.82% of observations (altogether 99.25% of the first data half were nearest neighbors to themselves in the full data). Notice that the consecutive contract days in the data (the follow-up time) were employee-specific, meaning that the outcome of ‘future’ SA days per year was aggregated over periods that differed between the employees. However, the unit “per year” (achieved via the offset term) is independent of the length of the aggregation period and each employee had ≥1.5 years of ‘future’ data and at least three years of data altogether.

### Tools for future research

Although we used halves of the original time series, the above-discussed algorithm for assigning cluster membership can be used to arbitrary new time series of a similar type. We release the codebooks based on our data together with our cluster-assignment algorithm to support further research. These are available online in the form of fiohpdc R package: https://bitbucket.org/rosenstroem/fiohpdc.

## Results

The permutation distribution clustering favored an eight cluster solution, as characterized in table 1 (absolute BIC difference > 127919 to other solutions). While some clusters had small number of employees, recall that each employee may entail up to 7845 numeric observations.

**Table 1:** The cluster sizes and averages for the eight detected clusters of working hours in employees of the Hospital District of Southwest Finland. [FIOH=Finnish Institute of Occupational Health; TM=employee’s time median value.]

Cluster	#1	#2	#3	#4	#5	#6	#7	#8
Number of employees (N)	289	55	893	151	4065	562	8	6
Men (%)	5.54	0.00	12.99	8.61	7.43	10.32	12.50	0.00
Contract days ^a^	4210.42	4119.58	3847.69	4222.47	2579.82	4124.01	4205.38	4067.33
Consecutive contract days ^b^	2343.63	2293.11	1916.23	2394.31	1053.38	2189.93	2428.25	2307.50
Age (years)	42.43	47.16	39.92	43.94	38.17	43.42	45.00	44.50
FIOH risk score for long work spells ^c^	0.19	0.09	0.19	0.08	0.15	0.17	0.03	0.03
FIOH risk score for night shifts ^c^	0.02	0.01	0.48	0.01	0.28	0.04	0.00	0.00
FIOH risk score for short recovery ^c^	0.26	0.01	0.35	0.01	0.22	0.03	0.02	0.24
TM work-shift length	7.97	7.98	8.25	7.98	8.26	7.98	8.00	8.00
TM length of rest hours	16.23	16.66	16.49	16.08	16.79	16.09	16.03	16.38
TM shift starting hours	7.44	7.73	9.89	7.56	8.96	7.70	8.36	8.50
Sickness absence days ^d^	8.58	11.35	11.76	5.81	13.03	11.62	9.67	5.59
Sickness absence trend ^e^	1.07	1.81	2.41 ^f^	2.36 ^f^	5.25 ^f^	3.18 ^f^	2.54	-3.16

a Contract days in our register sampling (employed days between dates 2008-01-01 to 2019-08-27)

b Work days in the longest consecutive stretch of data (without > 4-day interruptions in contract)

c Recommendation-based burden scores (range 0–3), with values growing with work overload (see supplementary material for exact units)

d Unit is a rate (days/year)

e Change from 1st to 2nd half of longest consecutive time series

f P <0.05.

### Cluster contents

**Figure 1:**
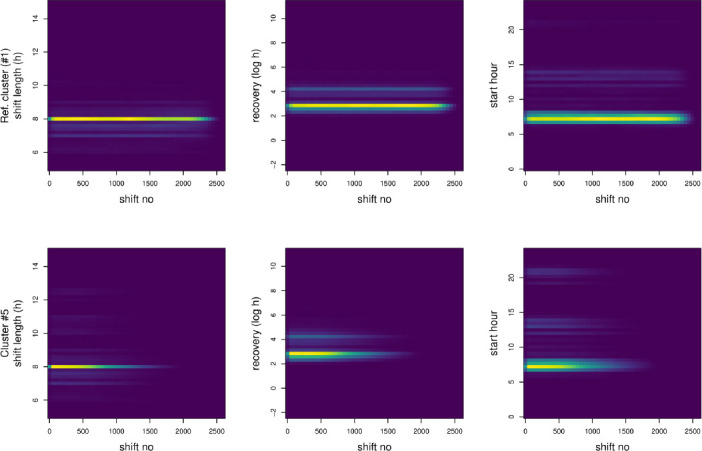
Empirical shift-by-characteristic densities for members of reference (ref.) cluster no 1 (upper row of panels; a panel per time-series dimension) and a risk cluster no 5 (lower row). Brighter colors indicate more probability mass. Note how individual shifts in the cluster #5 are more spread out on the vertical axes compared to cluster #1, and how the employees longest consecutive contract periods tend to end earlier on (ie, employees in cluster #5 have less work shifts overall). Similar figures on the other six clusters are available in the supplementary material online.

**Figure 2:**
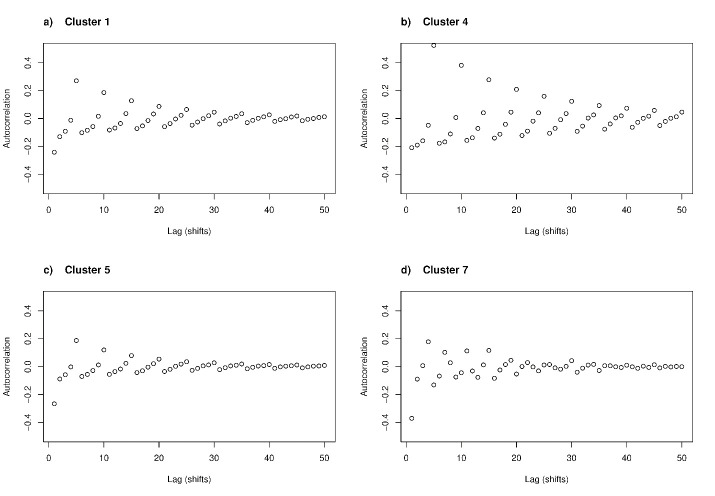
Cluster-average autocorrelation for log-recovery length. For each cluster, autocorrelation functions were computed for every employee and then averaged over the employees. Autocorrelation function indicates the correlation of a value at time t with the value at time t - l, where l is a time lag. Note how every 5th work shift tends to be strongly correlated in employees of cluster #4 (panel b), whereas the other clusters have either less pronounced weekly rhythm (clusters #1 and especially #5 in panels a and c) or a lack of clear 5-day rhythm (cluster #7 in panel d). Similar figures on the other four clusters are available in the supplementary material online.

In table 2, we provide our interpretations of the cluster contents for the convenience of the reader. However, this is not an exhaustive listing of their properties. The interpretations in table 2 arise from examining empirical shift-by-characteristic densities (cf. figure 1), average autocorrelations (cf. figure 2), basic descriptives (table 1), and examples of individual sequences of work shifts (the supplementary material shows more data characterizing the cluster contents). The distribution of between-shift rest periods was a long-tailed distribution containing many observations <24 hours but also occasional very long ‘rests’ (eg, holidays). In the figures, we therefore investigated logarithm of rest length: log-transformation retains relative order but shortens the tail (the assumption here is that eg, difference of 1 versus 2 months rest is less consequential than a difference of 1 versus 2 days).

**Table 2:** Working hour cluster interpretations in the hospital shift work data. [M=morning; E=evening; N=night].

Cluster	Short description ^a^	Typical characteristics
#1	Regular M/E work, weekends off	Regular morning and evening work with regular shift length, regular rest periods, and weekends off
#2	Irregular M work	Morning shifts with regular shift lengths, irregular rest periods, and weekend shifts
#3	Irregular M/E/N work	Three shift work (includes night shifts) with variable shift length, irregular rest periods, and weekend shifts
#4	Regular M work, weekends off	Regular morning work with regular shift lengths and rest periods, and weekends off
#5	Irregular, interrupted M/E/N work	Three shift work (includes night shifts) with variable shift length, irregular rest periods, weekend shifts, and short work contracts
#6	Variable M work, weekends off	Variable shift lengths and start times (regularly at mornings but irregular in exact timing), and weekends off
#7	Quickly rotating M/E work, non-standard weeks	Morning and evening work with regular shift lengths, weekend shifts, 4-day weeks, or alternating 3- and 5-day weeks, and many quickish ^b^ (<13 hours) returns to work
#8	Slowly rotating M/E work, non-standard weeks	Morning and evening work with regular shift length, variable days of week at work, and only few quickish (<13 hours) returns

a Some shifts do deviate from the typical pattern within clusters (“typical” merely highlights cluster differences)

b As an 11-hour return is typically called “quick”, we call a 13-hour return “quickish”

For example, cluster #5 clearly had more variable shift lengths and shift start hours than reference cluster #1 (figure 1). Compared to the other clusters, cluster #5 contained relatively short careers within the target employee population (table 1).

To further characterize differences between the clusters, we investigated the relative contributions of the three time series dimensions to the average distances between the clusters. For example, the dimension-wise average distances between clusters #1 and #5 were 1.76, 2.23, and 1.34 for the dimensions of work shift length, between-shift rest period, and start hour, respectively. In this sense, clusters #1 and #5 differed most in their between-shift rest periods. Similarly, the distances, eg, between clusters #1 and #7, were 1.67 (shift length), 2.69 (recovery), and 2.08 (start) while those of #7 and #8 were 1.07 (shift length), 2.57 (recovery), and 1.67 (start). These patterns suggest that largest differences between clusters involve between-shift rest periods.

To understand the between-shift rest periods permutation distributions beyond cluster-average distributions, figure 2 presents the cluster-average autocorrelations of between-shift rest periods. From figure 2, we observed that employees in cluster #1 followed on average a regular pattern where every fifth rest-length was strongly positively correlated, probably representing weekends. This pattern was even more pronounced in cluster #4, which turned out to be protective against sick leaves. The pattern was less pronounced in cluster #5 and missing from cluster #7. The latter cluster fitted better with a cycle where four work shifts are followed by a longer rest and then three work shifts. Thus, some clusters (eg, #5) were associated with less predictable work-rest cycles than others (eg, #1), but not necessarily with much less between-shift rest periods or a better FIOH recovery risk score (cf. table 1).

The small clusters – #7 and #8 – also differed in their marginal distributions for rest hours. Whereas the FIOH risk scores indicated similar levels of quick returns assessed in < 11-hour rests (table 1), 32.4% of shifts in cluster #7 were followed by <13 hours of rest – significantly more than the 14.48% in cluster #8.

Although clusters #4 and #6 were similar to each other in many respects, an examination of individual employees shift patterns led to a hypothesis that cluster #6 had much more variable exact work shift lengths and starting times than cluster #4 (despite both involving morning shifts).

### Associations between clusters and sickness absence

Table 3 presents the associations between the work shift patterns and SA. We chose cluster #1 as a reference cluster because it represented a comparatively large cluster of regular, *a priori* non-risk working hour conditions, and had no SA trend (table 1). In general, the clusters were associated with SA rates over and above (adjusting for) sex and age (Model 1, table 3). The clusters were also associated with SA when adjusting for sex, age, and the three FIOH risk scores on working hours (Model 2, table 3), and also while controlling for historical employee-specific SA rates (Model 3, table 3).

**Table 3:** Multiple Poisson regression analyses on associations between working hour clusters and sickness absence rates. For the categorical variables, the incidence rate ratio (IRR) value implies an IRR-fold number of sickness absence (SA) days per year compared to the reference category. For comparability with the categorical effects, a two standard deviation difference in a continuous variable (age or past SA) implies an IRR-fold number of SA days per year; (cf. 26). M/E/N work refers to presence of morning, evening, and night work; M/E to primarily morning and evening work; and M to morning work only. [IRR=incidence rate ratio; CI=confidence interval; FIOH=Finnish Institute of Occupational Health].

Variable set	Model 1 ^a^ (cross-sectional)		Model 2 ^b^ (cross-sectional)		Model 3 ^c^ (longitudinal)	
		
	IRR	95% CI	IRR	95% CI	IRR	95% CI
Demographic						
Woman (reference=men)	1.01	1.00–1.02	0.97	0.96–0.98	0.90	0.88–0.91
Age	1.38	1.37–1.38	1.39	1.38–1.39	1.36	1.35–1.37
FIOH risk scores						
Long work spells			0.82	0.81–0.82	0.98	0.98–0.99
Night shift burden			1.01	1.00–1.02	0.99	0.99–1.00
Recovery burden			1.01	1.01–1.02	1.02	1.01–1.03
Working hour clusters						
#1 Regular M/E work, weekends off (reference)	1.00		1.00		1.00	
#2 Irregular M work	1.25	1.21–1.28	1.17	1.14–1.20	1.20	1.15–1.24
#3 Irregular M/E/N work	1.44	1.42–1.46	1.43	1.41–1.45	1.35	1.32–1.37
#4 Regular M work, weekends off	0.67	0.65–0.68	0.62	0.61–0.64	0.83	0.80–0.85
#5 Irregular, interrupted M/E/N work	1.65	1.63–1.67	1.61	1.59–1.63	1.77	1.74–1.80
#6 Variable M work, weekends off	1.35	1.33–1.37	1.33	1.32–1.35	1.35	1.32–1.38
#7 Quickly rotating M/E work, non-standard weeks	1.10	1.03–1.17	0.99	0.93–1.06	1.11	1.02–1.22
#8 Slowly rotating M/E work, non-standard weeks	0.65	0.59–0.72	0.59	0.53–0.65	0.44	0.37–0.52
Past sickness absence (days/year standardized)					1.64	1.63–1.64

a Model 1 predicts all sickness absences.

b Model 2 further adjusts Model 1 for FIOH recommendations.

c Model 3 predicts future sickness absence adjusting for past employee-specific sick leaves.

Working hour clusters #1, #4, and #8 were associated with the lowest SA rates. Cluster #7 implied an elevated risk. Cluster #5 implied the highest risk among all the covariates, followed by clusters #3 and #6.

## Discussion

In this study of 6029 hospital employees in mainly full-time shift work during a 12-year follow up, we identified eight distinct working hour patterns in a data-driven clustering analysis of the entire work-time series. These patterns ranged from relatively regular morning- and evening-oriented shift work with weekends off (cluster #1) to highly irregular working hours with night and weekend shifts under short contracts (cluster #5). The clusters associated with unpredictable or irregular working hours, short between-shift rest periods, or short contracts (clusters #2, #3, #5, #6, #7) were associated with greater risk of future SA than clusters lacking these features (clusters #1, #4, and #8). Cluster #5, which combined these features, was most strongly associated with the risk of SA.

In general, our data-driven strategy corroborated previous hypothesis-based research on the associations of working hour characteristics with SA risks. For example, quick returns, irregular working hours and night and weekend shifts were more common in clusters associated with high SA rates (11, 16). It was also clear both quantitatively and qualitatively (see below) that the comprehensive data clusters captured novel risk characteristics that are not usually identified when analyzing pre-defined working hour characteristics separately. For example, cluster effects on SA rate were comparable to predicting SA with earlier SA while also withstanding an adjustment for earlier SA. This is noteworthy as history of SA is a known strong predictor of SA rate (27, 28). Furthermore, FIOH risk scores, based on a traditional hypothesis-driven approach, offered little information on SA beyond the clusters. The next paragraph outlines other new insights.

First, both clusters #3 and #5 could be characterized by the above high-risk working hour characteristics, but cluster #5 implied even greater risk of SA than cluster #3 – even though the reverse should have happened, if anything, based on associations with the earlier risk scores (table 1). However, the employees in cluster #5 had shorter and more gapped contracts than those in #3. The career interruptions may have been caused by SA (implying reverse causation), but they may also cause job insecurity and relational injustice that might have complex effects on SA rate (29). Future studies of work hour characteristics and SA might benefit from considering contract length and form (temporary versus permanent).

Second, based on broad qualitative characterizations it might have been difficult to tell apart the employees in clusters #4 and #6, both of which were associated with morning-oriented work with free weekends (table 2). Yet, the data-driven clustering method alerted us to some difference between clusters 4 and 6 and, indeed, cluster #6 was associated with an increased risk of future SA and cluster #4 with a decreased risk. We then observed that cluster #6 contained more variation in shift length and starting time *within* the set of morning shifts than cluster #4. This suggests presence of either an internal or an external factor that hinders the employees settling on an accurate work routine, which may contribute to SA. Thus, future studies could be conducted on the fine-grained variations in morning-oriented shift schedules and related factors. For example, employee possibility to control working hours via participatory working time scheduling can decrease SA and presumably alter working hour patterns (30).

Third, previous research has investigated quick returns (<11 hours), probably because that has been the minimum daily rest in European Working Time Directive (11) and, yet, our data-driven analysis uncovered groups (clusters #7 and #8) that had negligible difference in quick returns, while having over two-fold differences in <*13-hour* returns and SA risk. This suggests that 13 hours between-shift rest period (ie, rest hours) might be a threshold for recovery for employees in hospital care. Future studies could question the typical threshold of quick returns (<11 hours) and instead consider a wider range of rest periods. These examples suggest how data-driven investigations could increase accuracy and coverage of research in the field by addressing ‘unknown unknowns’ (by exploration), not just ‘known unknowns’ (*a priori* hypotheses).

Our study presented an investigation of register-based working-hour time series in relation to health that was data-driven and hypothesis-free, to an unprecedented degree to our knowledge. Besides that, the strengths of the study were a large amount of reliable (objective, payroll-based) register data on the real-world working hour characteristics of shift work and SA in hospitals, a robust and general clustering procedure, and adjustments for both historical SA and previous risk scores.

Limitations of our study include it being an observational data-driven study with the associated limitations to causal inference, and possible limited generalizability due to having data from just one, albeit large, hospital district in Finland. For example, in some of our clusters, the number of men was low, although that is generally in line with female-dominated sex distribution in healthcare sector (31). As people with particular socioeconomic and health profiles may select into particular shift work schedule, future studies should address unmeasured confounders, such as having children. Those researchers who have been able to measure such variables could use our *fiohpdc* package to assess their possible relationships with our clusters (see Methods section). One might also further gauge the generalizability of clusters #7 and #8 that, while reflecting tens of thousands of sequential numeric observations, nevertheless had only few employees in our data. In addition, data-analytic approaches like the present one are frequently limited by their many degrees of freedom for the researchers. For example, besides BIC, we also tested Akaike’s information criterion for selecting the appropriate number of clusters, but that would have led to very many small clusters and therefore BIC appeared to us as a principled and pragmatic choice (25). Nevertheless, there remains scope for investigating alternative cluster solutions, and alternative time series clustering methods, such as methods using autocorrelation-based or Maharaj distances (21, 22). We hope the present work inspires others through our released cluster assignment package that allows others to map their data on our cluster as closely as possible (see Methods).

In summary, our data-driven cluster analysis identified eight different clusters of working hour characteristics in shift work among hospital employees. The identified shift work clusters were associated with the rate of SA after adjusting for previous SA and previously suggested risk scores. The strongest risk of SA was associated with highly irregular working hours with night and weekend shifts and interrupted job contracts.

## Supplementary material

Supplementary material
