# Supplementary material for: Patterns of working hour characteristics and risk of sickness absence among shift-working hospital employees: a data-mining cohort study
Source: Scand J Work Environ Health. 2021 Jun 29;47(5):395–403. doi: 10.5271/sjweh.3957 (PMC8259704; doi:10.5271/sjweh.3957)
Supplement: Supplementary material [file SJWEH-47-395-S001.pdf]

SUPPLEMENTARY MATERIAL: Patterns of working hour characteristics and risk of sickness absence in shift working hospital employees: a data-mining cohort study

Contents

Appendix 2
Work shift burdensomeness in FIOH shift ergonomics 2
Supplementary information on cluster correlates and contents 2
Supplementary Tables: Correlates for cluster memberships 3
Supplementary Table S1 3
Supplementary figures characterizing cluster contents 5
Supplementary Figure S1 5
Supplementary Figure S2 6
Supplementary Figure S3 7
Supplementary Figure S4 8
Examples of Shift Rotations 9
Weekdays 9
Raw-data Plots 11

Appendix

Work shift burdensomeness in FIOH shift ergonomics

In the main text, we compared our novel data-driven clusters to more traditional work overload risk scores derived from past literature by an expert panel. The work overload risk scores in the FIOH (Finnish Institute of Occupational Health) shift ergonomics recommendations were formed according to the following rules:

Recommendation content	Score 3	Score 2	Score 1	Score 0
Working hours between 2 free days (h)	> 55 h	48:01-55:00	40:01-48:00	≤ 40
———————————————	———	————-	————-	———-
Number of night shifts in 3 weeks	≥ 9	5–8	3–4	0–2
———————————————	———	————-	————-	———-
Quick (<11 h) returns in 3 weeks	≥ 9	5–8	3–4	0–2

A shift with at least 3 hours of work between 23–06 o'clock was considered a night shift. The full set of recommendations have been published in Finnish under https://www.ttl.fi/tyoaika (14). Direct link here. Note, the FIOH recommendations are distinct from our data-driven clusters and here they were used merely as a point of comparison.

In this study, each possible period that allowed the calculation of the above scores for a given employee was used to do so. The numbers were then averaged to find an employee-specific risk value (range 0–3) for the target interval (e.g., for first half of his or her longest consequtive data stretch).

Supplementary information on cluster correlates and contents

Description of clusters in the main text was necessarily limited due to the space constraints and for the sake of brevity. In below, we supplement the description. First, we provide correlates of individual clusters by regressing them on multiple covariates (Table S1), although this remains a linear characterization with limited value for the minor clusters. Second, we provide figures used to characterize the clusters in terms of their input data also for the clusters that could not be illustrated in the main text due to the space constraints (Figure S1–S4). Finally, to understand the highly non-parametric cluster model, it is useful to investigate some individual members of the clusters. For that purpose, the remaining text shows the weekdays of the first 25 shifts in the first 6 members (employees) in each of the 8 clusters, whereas the remaining figures (raw data plots) show the quantitative dimensions of shift length, rest length, and shift start time for the first 100 shifts of the first 4 members in each of the 8 clusters.

Supplementary Tables: Correlates for cluster memberships

In the main text, we adjusted effects of our data-driven clusters on sickness absence for other variables. Below, and the reader may find how those other variables are associated with the clusters themselves.

Supplementary Table S1. **Multiple logistic regressions predicting specific cluster membership against the other clusters with standardized variables (OR = odds ratio, CI = confidence interval).**

Predictor variable	Cluster #1		Cluster #2		Cluster #3		Cluster #4	
	OR	95% CI	OR	95% CI	OR	95% CI	OR	95% CI
(Intercept)	1.05***	1.04–1.05	1.01***	1.01–1.01	1.16***	1.15–1.17	1.03***	1.02–1.03
Man	1.02*	1.00–1.04	1.01*	1.00–1.02	0.94***	0.92–0.97	1.00	0.98–1.01
Contract days[a]	0.97***	0.95–0.98	0.99*	0.98–1.00	1.03*	1.00–1.06	0.98***	0.97–1.00
Consecutive contract days[b]	1.20***	1.17–1.22	1.04***	1.03–1.04	1.30***	1.26–1.34	1.10***	1.09–1.12
Age	0.99	0.98–1.00	1.01*	1.00–1.01	1.01	0.99–1.03	1.00	0.99–1.01
FIOH[c] work spells burden	1.02***	1.01–1.03	0.99**	0.99–1.00	0.98*	0.96–1.00	0.97***	0.96–0.98
FIOH night shift burden	0.96***	0.94–0.98	1.00	0.99–1.01	1.23***	1.19–1.27	1.00	0.98–1.01
FIOH recovery burden	1.04***	1.03–1.05	0.99*	0.99–1.00	1.16***	1.14–1.18	0.97***	0.96–0.98
TM[d] work-shift length	1.02**	1.01–1.03	1.00	0.99–1.01	0.97**	0.95–0.99	1.01	1.00–1.02
TM length of rest hours	1.01	1.00–1.02	1.00	1.00–1.01	1.01	0.99–1.02	1.00	0.99–1.01
TM shift starting hours	0.99	0.97–1.01	1.00	1.00–1.01	0.96*	0.94–0.99	1.01	0.99–1.02
Sickness absence days[e]	0.98***	0.97–0.99	1.00	0.99–1.00	1.01	1.00–1.03	0.97***	0.96–0.98
Sickness absence trend[f]	1.00	0.99–1.01	1.00	0.99–1.00	0.99	0.98–1.00	1.01*	1.00–1.01
	Cluster #5		Cluster #6		Cluster #7		Cluster #8	
	OR	95% CI	OR	95% CI	OR	95% CI	OR	95% CI
(Intercept)	1.96***	1.95–1.98	1.10***	1.09–1.1	1.00**	1.00–1.00	1.00*	1.00–1.00
Man	1.05***	1.02–1.08	0.98	0.96–1.01	1.00	1.00–1.00	1.00	1.00–1.00
Contract days[a]	1.03*	1.00–1.06	1.00	0.98–1.03	1.00	0.99–1.00	1.00	1.00–1.00
Consecutive contract days[b]	0.45***	0.44–0.46	1.25***	1.21–1.28	1.01***	1.00–1.01	1.00**	1.00–1.01
Age	0.99	0.98–1.01	1.00	0.98–1.01	1.00	1.00–1.00	1.00	1.00–1.00
FIOH[c] work spells burden	1.02	1.00–1.03	1.03***	1.01–1.04	1.00*	1.00–1.00	1.00*	1.00–1.00
FIOH night shift burden	0.88***	0.86–0.91	0.97*	0.94–1.00	1.00	0.99–1.00	1.00	0.99–1.00
FIOH recovery burden	0.97***	0.95–0.98	0.89***	0.88–0.9	1.00	1.00–1.00	1.00	1.00–1.00
TM[d] work-shift length	1.02*	1.00–1.04	0.98*	0.96–1.00	1.00	1.00–1.00	1.00	1.00–1.00
TM length of rest hours	0.98*	0.97–1.00	1.00	0.99–1.02	1.00	1.00–1.00	1.00	1.00–1.00
TM shift starting hours	1.01	0.99–1.04	1.02	0.99–1.04	1.00	1.00–1.01	1.00	1.00–1.01
Sickness absence days[e]	1.03***	1.02–1.05	1.01	0.99–1.02	1.00	1.00–1.00	1.00	1.00–1.00
Sickness absence trend[f]	1.00	0.99–1.01	1.01	0.99–1.02	1.00	1.00–1.00	1.00	1.00–1.00

*** $p < 0.001$; ** $p < 0.01$; * $p < 0.05$; See ref. 26 in main text for predictor standardization.

[a] Contract days in our register sampling (employed days between dates 2008-01-01 to 2019-08-27)

[b] Work days in the longest consecutive stretch of data (without > 4-day interruptions in contract)

[c] FIOH = Finnish Institute of Occupational Health recommendation-based burden scores (range 0–3), with values growing with work overload (see Appendix for exact units)

[d] TM = employee's time median value

[e] Unit is a rate (days/year)

[f] Change from 1st to 2nd half of longest consecutive time series (*** for $p < 0.001$; ** for $p <0.05$)

Supplementary figures characterizing cluster contents

In the main text, we showed figures characterizing the cluster contents, for some key clusters only for brevity. For the sake of comprehensiveness, the below supplementary figures show the same information for the remaining clusters.

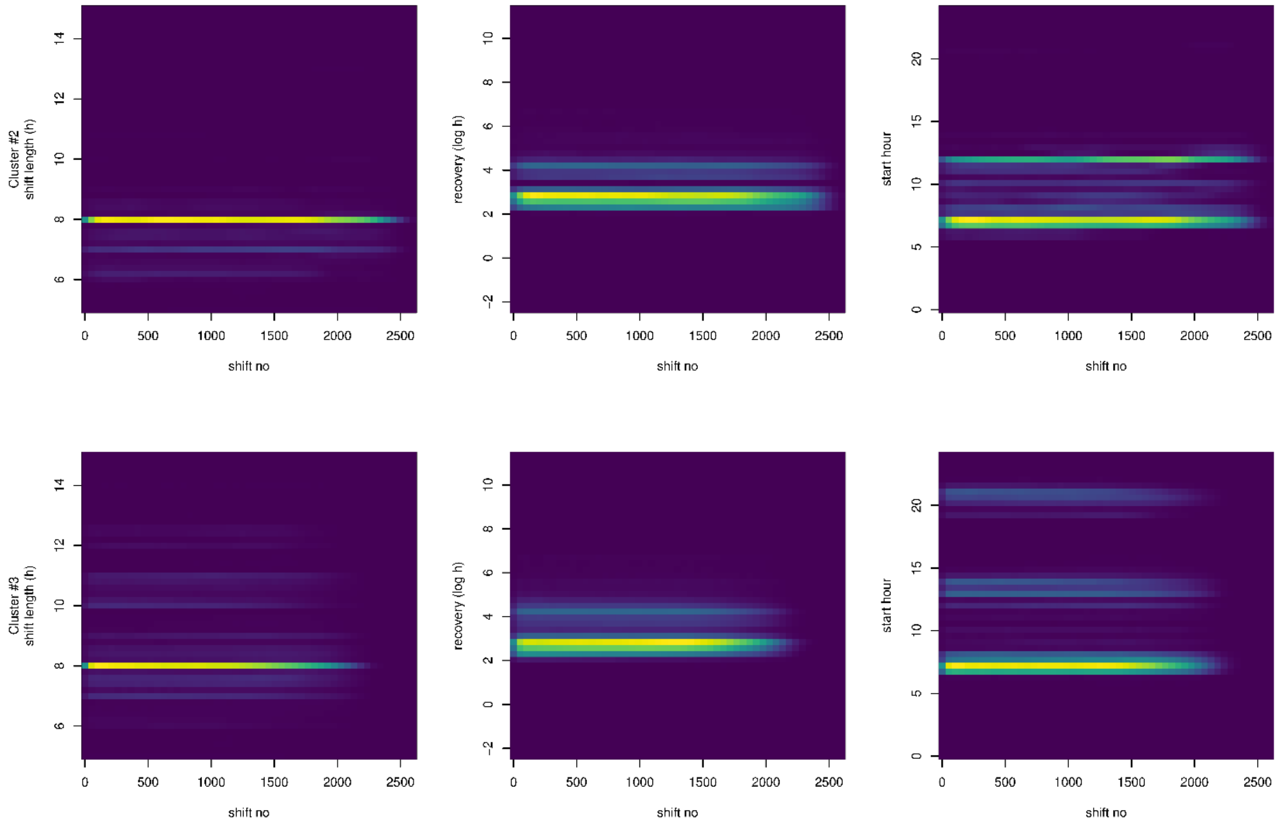

Supplementary Figure S1. Empirical shift-by-characteristic densities for cluster no 2 (upper row) and cluster no 3 (lower row). Brighter colors indicate more probability mass.

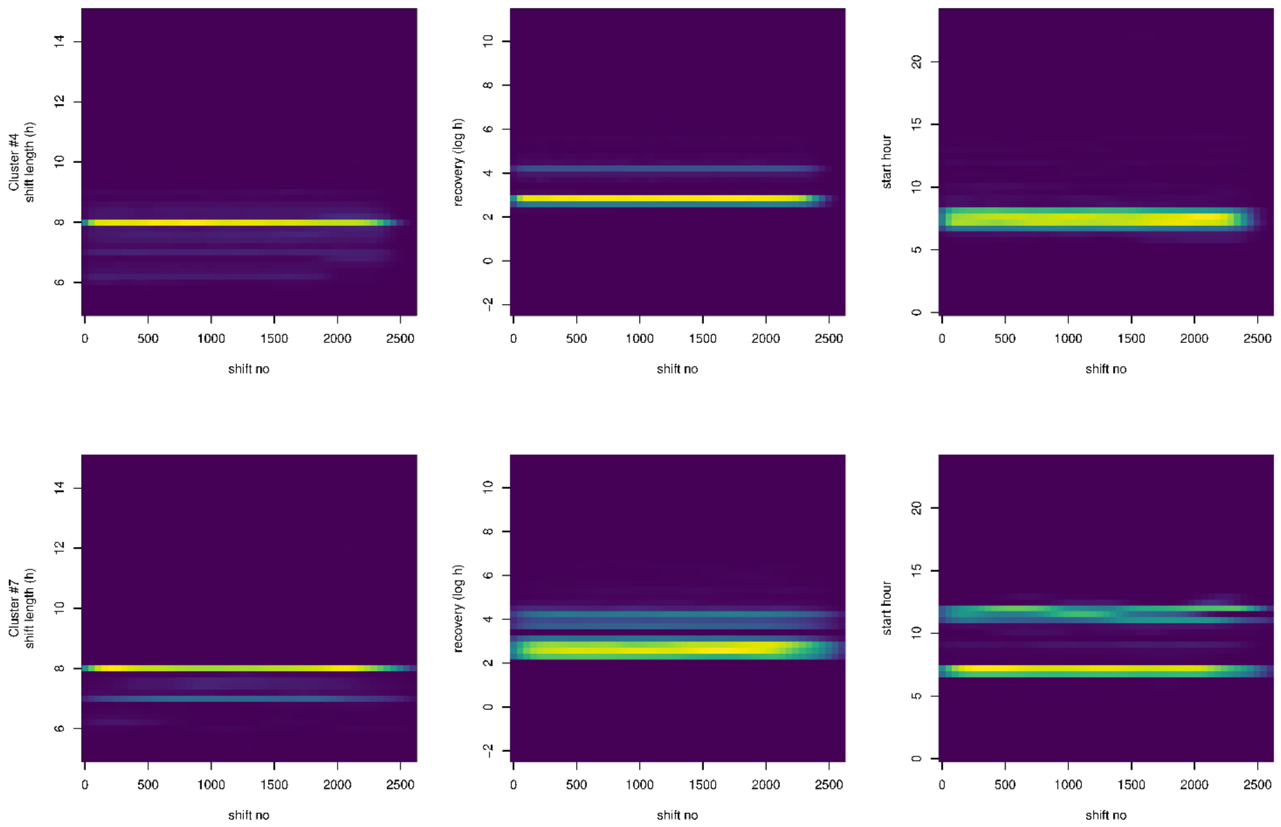

Supplementary Figure S2. Empirical shift-by-characteristic densities for cluster no 4 (upper row) and cluster no 7 (lower row). Brighter colors indicate more probability mass.

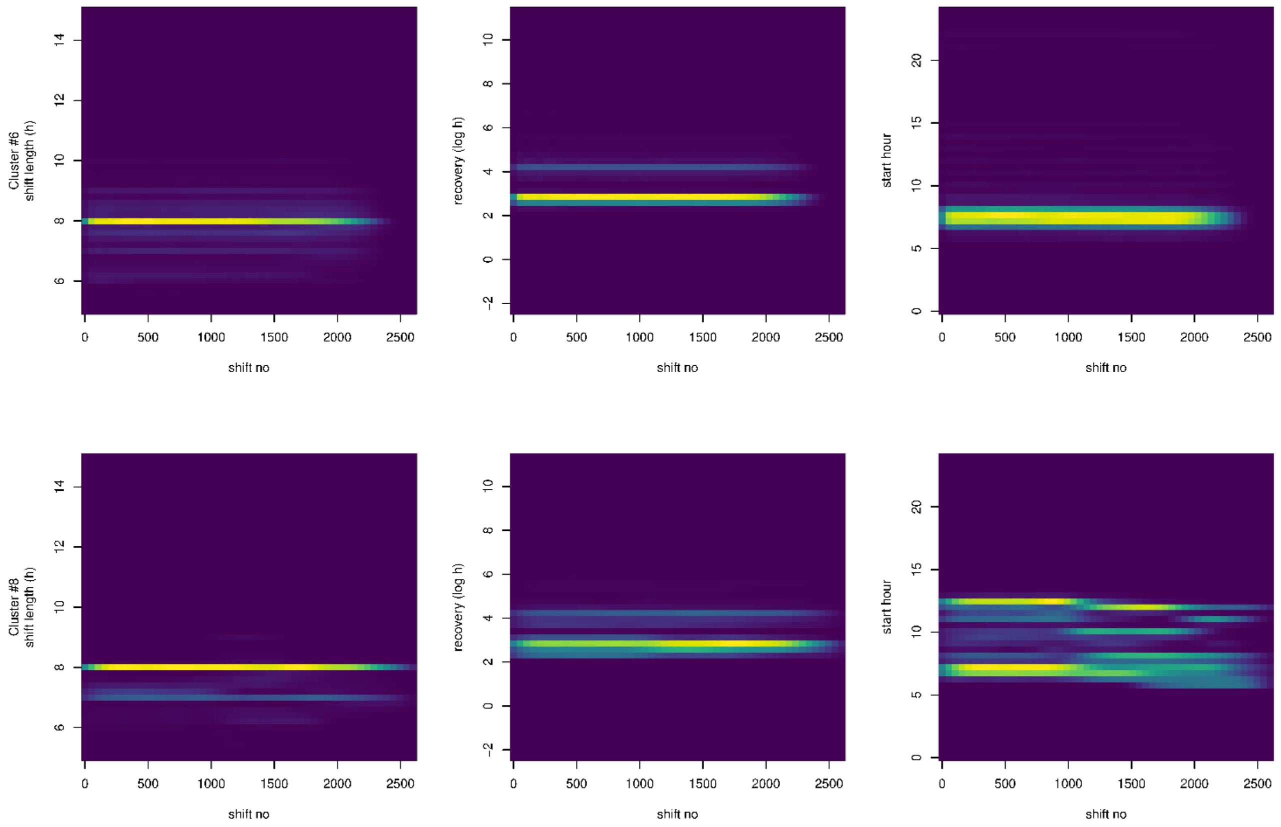

Supplementary Figure S3. Empirical shift-by-characteristic densities for cluster no 6 (upper row) and cluster no 8 (lower row). Brighter colors indicate more probability mass.

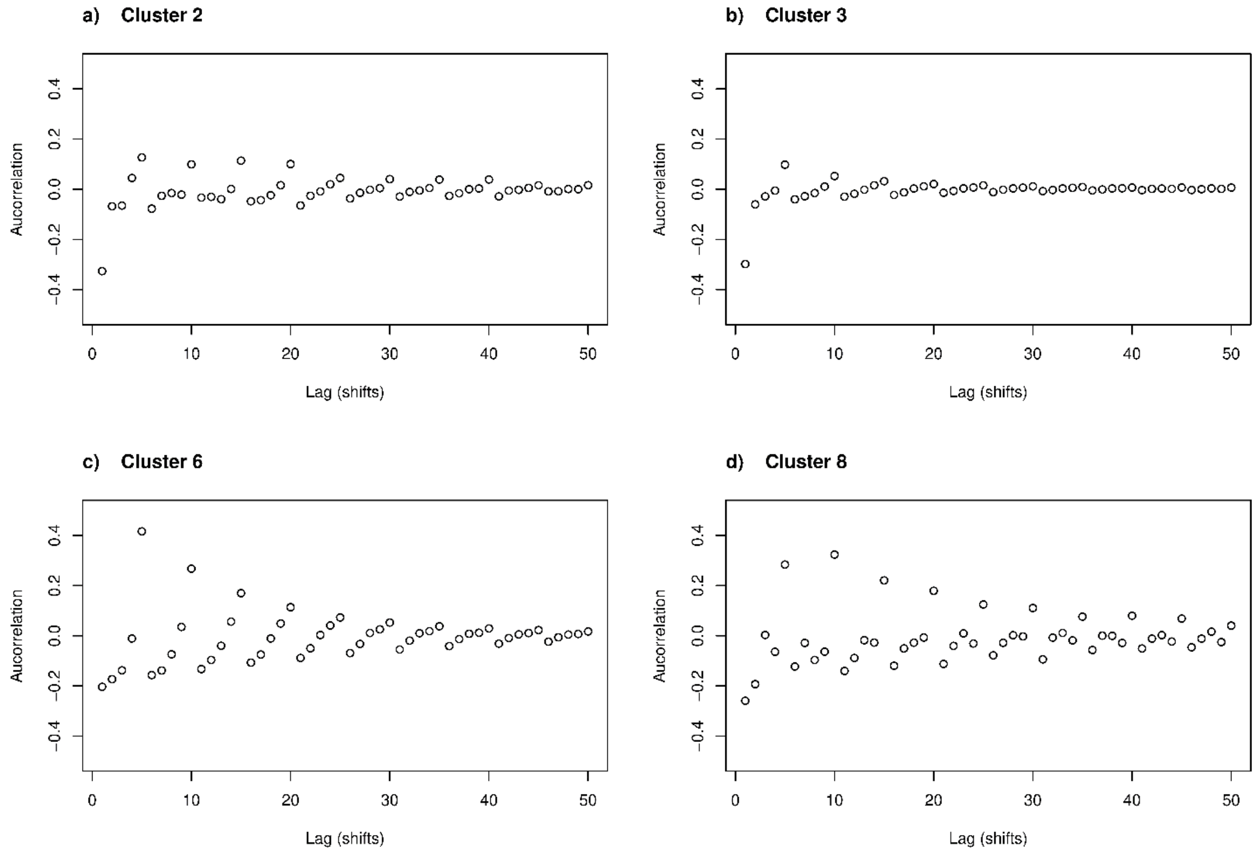

Supplementary Figure S4. Cluster-average autocorrelation for log-recovery length in clusters not shown in the main text.

Examples of Shift Rotations

Weekdays

In the below print out, weekdays of successive shifts are shown for 6 first samples in each cluster (mo = Monday; tu = Tuesday; we = Wednesday; th = Thursday; fr = Friday; sa = Saturday; su = Sunday).

```
## "Examples of rotations: first 25 shifts in ..."

## "Six cluster 1 examples:"
##  [1] mo tu we th fr mo tu we th fr mo tu we th fr mo tu we th fr th fr mo tu we

##  [2] we th fr mo tu we th fr mo th fr mo tu we th fr mo tu th fr sa mo tu we th

##  [3] tu we th fr tu we th su mo we th fr sa su mo th fr sa su mo tu th fr tu we

##  [4] tu th fr mo tu we th fr mo tu we fr sa su mo we th mo tu we th fr mo tu we

##  [5] tu mo tu fr sa mo tu tu we th fr su sa su mo th fr sa su mo tu we mo tu we

##  [6] tu we th fr tu we th fr sa we th fr sa su mo tu sa su mo tu we th we th fr

## "Six cluster 2 examples:"
##  [1] mo tu we th fr mo tu we th sa su tu we th fr mo tu we th fr mo tu we th fr

##  [2] fr sa su mo we th fr sa mo tu we th fr tu we th sa su mo tu fr sa su mo we

##  [3] we fr sa su we th fr sa mo tu we th fr tu we th sa su mo tu fr sa su mo we

##  [4] tu we th mo tu we th tu we th fr sa su mo th fr sa mo tu we mo tu we th fr

##  [5] tu we su mo mo tu we th sa su mo tu th fr sa tu we th fr sa su tu we th fr

##  [6] we th fr mo tu we fr mo tu we fr mo we th fr we th fr mo tu we th fr mo mo

## "Six cluster 3 examples:"
##  [1] mo tu we th fr mo tu we th fr mo tu we th fr mo tu we th fr mo tu we th fr

##  [2] we th fr su mo th fr sa tu we th su mo th fr su sa su th fr sa mo tu we th

##  [3] mo tu we th sa tu we th fr mo tu we th fr mo tu we fr mo tu we we th fr mo

##  [4] tu mo tu we th fr mo tu we th fr mo tu we th fr mo tu th fr mo tu we th fr

##  [5] we sa su mo tu we th fr mo tu we th fr mo tu we sa su mo tu we mo tu we th

##  [6] sa su mo tu sa su tu we su mo tu we th tu we th fr sa we th fr we th fr sa

## "Six cluster 4 examples:"
```

```
##  [1] mo tu we th fr mo tu we fr mo tu we th fr mo tu we th fr mo tu we th fr mo

##  [2] we th fr mo tu we th fr mo tu we th fr mo tu we th fr mo tu we th fr mo tu

##  [3] we th fr mo tu we th fr mo tu we th fr mo tu we th fr mo tu we th fr mo tu

##  [4] we th fr mo tu we th fr mo tu we th fr mo tu we th mo tu we th fr mo tu we

##  [5] we th fr mo tu we th fr mo tu we th fr mo tu we th fr mo tu we th fr sa mo

##  [6] fr sa su th fr mo tu th fr sa mo tu we th fr su mo tu we th fr tu we th fr
```

"Six **cluster 5** examples:"

```
##  [1] tu we th fr mo we fr sa mo tu sa su mo tu we th sa su mo tu th fr sa tu we

##  [2] fr sa mo tu we th fr mo tu th fr mo tu we th sa su mo tu we th fr mo tu we

##  [3] fr mo tu we th fr mo tu we th fr mo tu we th fr mo tu we th fr mo tu we th

##  [4] su mo tu we mo tu fr sa su mo tu we tu we fr sa su mo sa su mo tu we tu we

##  [5] mo tu th fr sa su mo tu sa su mo we th tu we th fr sa su th fr mo tu fr mo

##  [6] mo tu we th fr mo tu we th fr mo tu we th fr mo tu we th fr mo mo tu we th
```

"Six **cluster 6** examples:"

```
##  [1] we th fr mo tu we th fr mo tu we th fr mo tu we th fr mo tu we th fr mo we

##  [2] mo tu we th fr mo tu we th fr mo tu we th fr mo tu we th mo tu we th fr mo

##  [3] we th fr mo tu we th fr mo tu we th fr mo tu we th fr mo tu we th fr mo tu

##  [4] mo tu we th fr mo tu we th fr mo tu we th mo tu we th fr mo tu we th mo we

##  [5] tu we th fr mo tu we th fr fr mo tu we fr mo tu we tu we th fr mo tu we th

##  [6] we th fr mo tu we th fr mo tu we th fr mo tu we th fr mo tu we th fr mo tu
```

"Six **cluster 7** examples:"

```
##  [1] tu we th fr sa th fr we th fr sa su tu we th fr sa su mo th fr mo tu we th

##  [2] we th fr sa su mo tu we fr sa su th fr sa su tu we th fr mo tu we fr sa su

##  [3] tu fr sa su we th fr mo tu we th sa su tu we th fr mo tu we th sa su mo tu

##  [4] we mo tu we sa su mo fr sa su tu we th mo mo th fr sa su tu we th fr mo tu

##  [5] tu we fr sa su tu we th fr mo tu we fr sa su mo th fr sa su tu we th fr mo

##  [6] tu we th mo fr sa su tu we th fr mo tu we th sa su mo tu we th fr tu we th
```

"Six **cluster 8** examples:"

```
##  [1] we th fr mo tu we th fr th fr mo tu we th fr mo tu we th fr mo tu we th fr
```

```
## [2] tu we fr mo tu we th mo tu we fr sa mo tu we th fr mo tu we fr sa mo tu we

## [3] we th fr mo tu we th fr mo tu we th mo tu we th fr mo tu we th fr mo tu we

## [4] we th fr su mo tu we th mo tu th fr sa su mo we th fr mo tu we th sa su mo

## [5] we th fr mo tu mo tu th fr sa su tu we th fr mo tu we th fr mo tu we th fr

## [6] mo tu we th fr mo tu we th fr mo tu we th fr mo tu we th fr mo tu we th fr
```

Raw-data Plots

The following plots show the data for the first 100 shifts for the first 4 employees in each cluster. The data are almost “raw” in the sense that registered values are plotted for the data dimensions entered into the permutation distribution clustering method. They are only “almost” because a logarithm was taken from the rest-length values for the sake of data-point distinguishability (some very large values present in the data). A dashed line in those panels was drawn at log(11h) ≈ 2.4 log-hours to highlight which recovery lengths are shorter than the minimum daily rest in the European Working Time Directive (see main text). Each figure contains an upper-left panel plotting shift lengths against calendar days. The adjacent plot shows the same against the running number of shifts, and the remaining plots the two other dimensions entering into the 3-dimensional time-series clustering (i.e., rest length and shift start time).

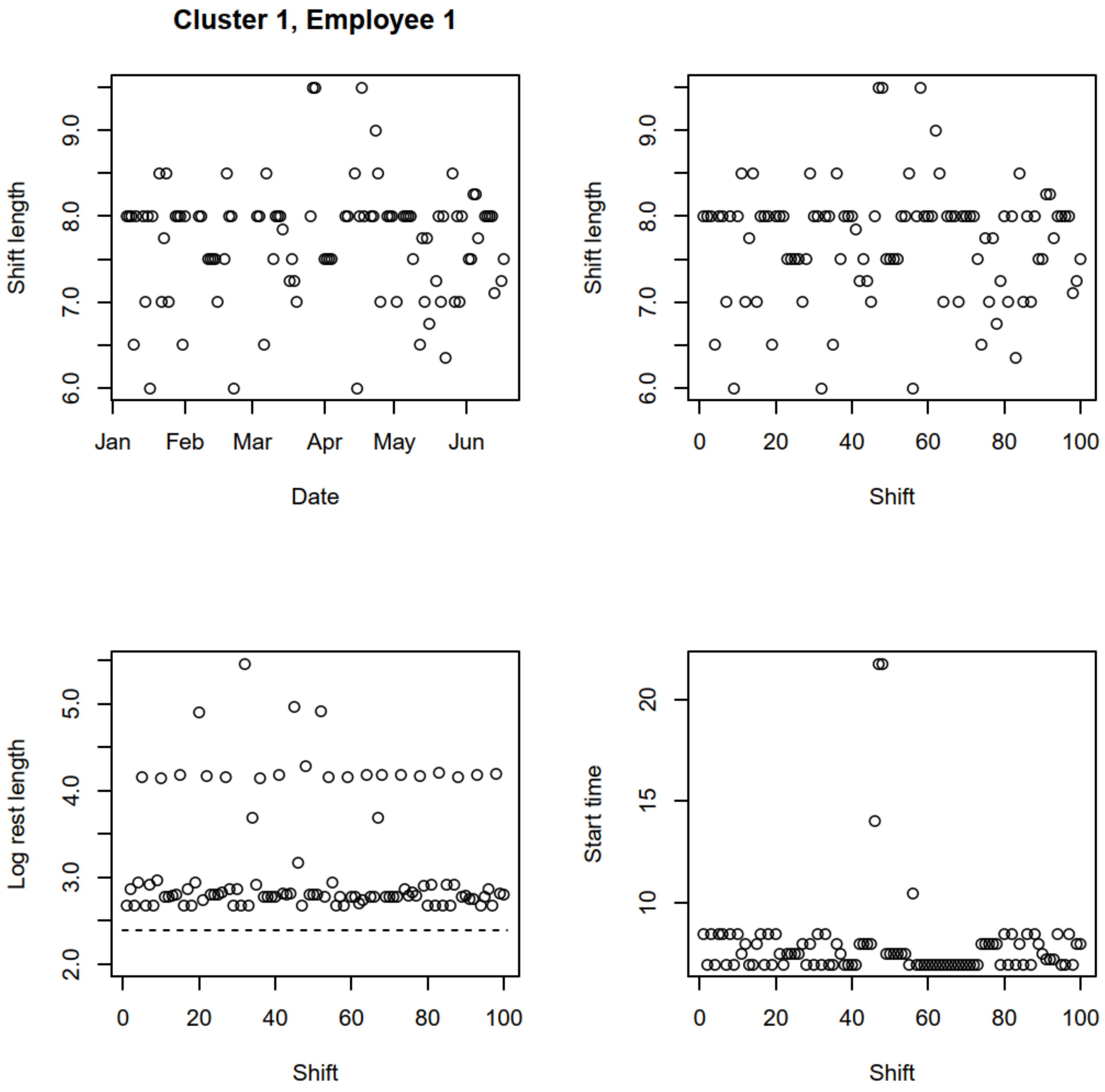
Cluster 1, Employee 1
Shift length
9.0
8.0
7.0
6.0
Jan
Feb
Mar
Apr
May
Jun
Date
Shift length
9.0
8.0
7.0
6.0
0
20
40
60
80
100
Shift
Log rest length
5.0
4.0
3.0
2.0
0
20
40
60
80
100
Shift
Start time
20
15
10
0
20
40
60
80
100
Shift

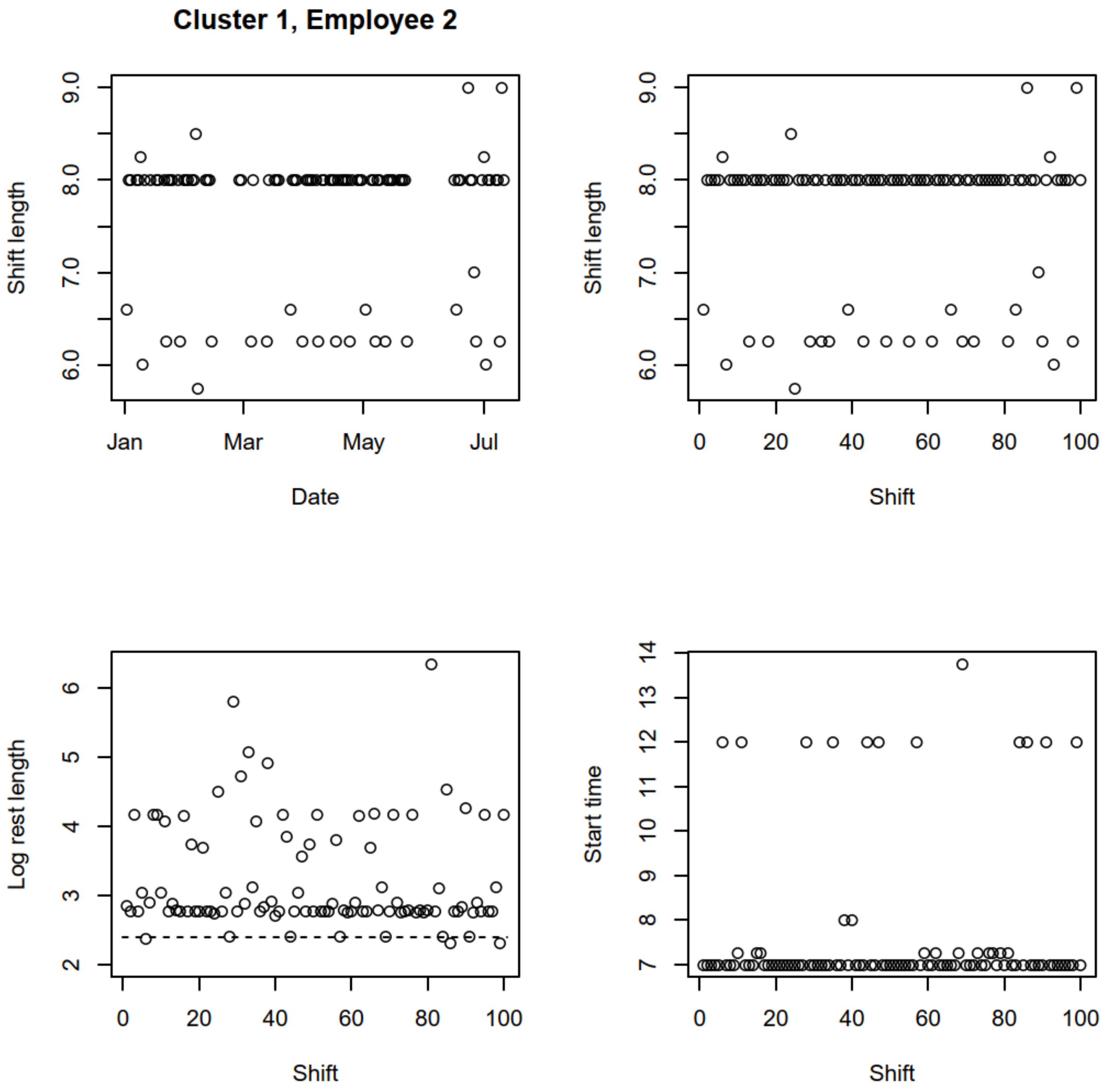
Cluster 1, Employee 2
Shift length
Date
Jan
Mar
May
Jul
Shift length
Shift
Log rest length
Shift
Start time
Shift

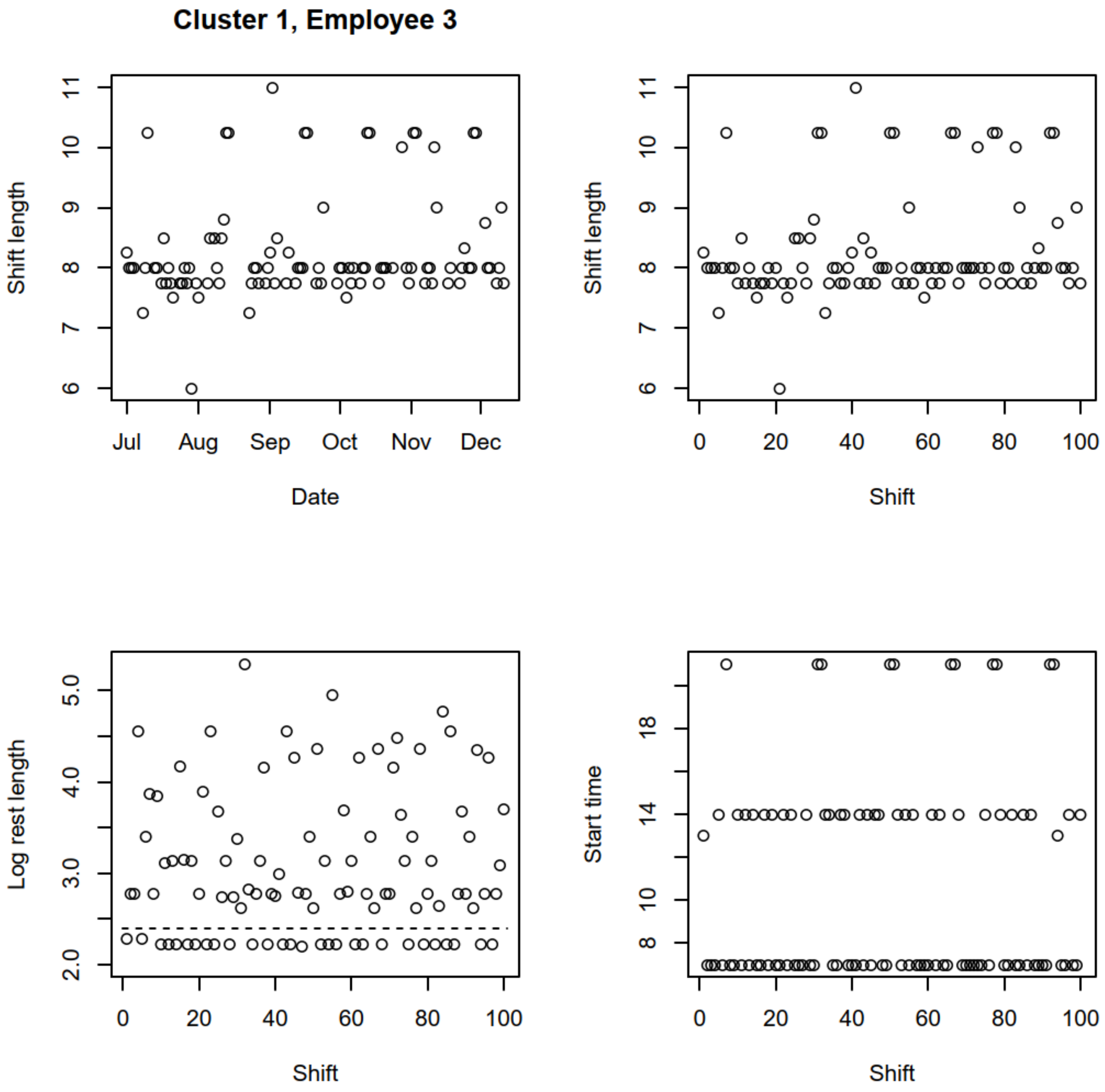
Cluster 1, Employee 3
Shift length
Date
Jul
Aug
Sep
Oct
Nov
Dec
Shift
Log rest length
Start time

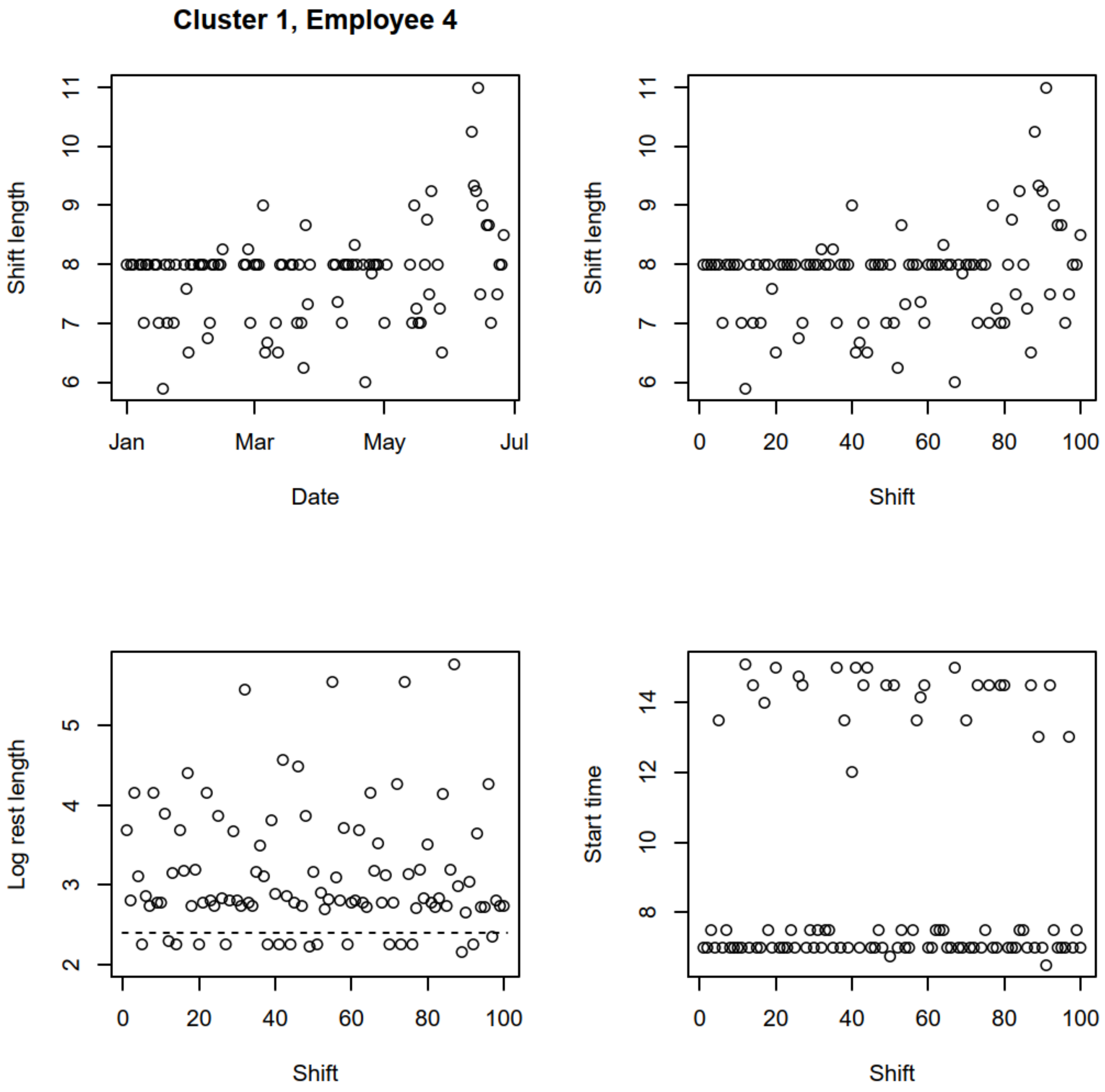
Cluster 1, Employee 4
Shift length
Date
Jan
Mar
May
Jul
Shift
Log rest length
Start time

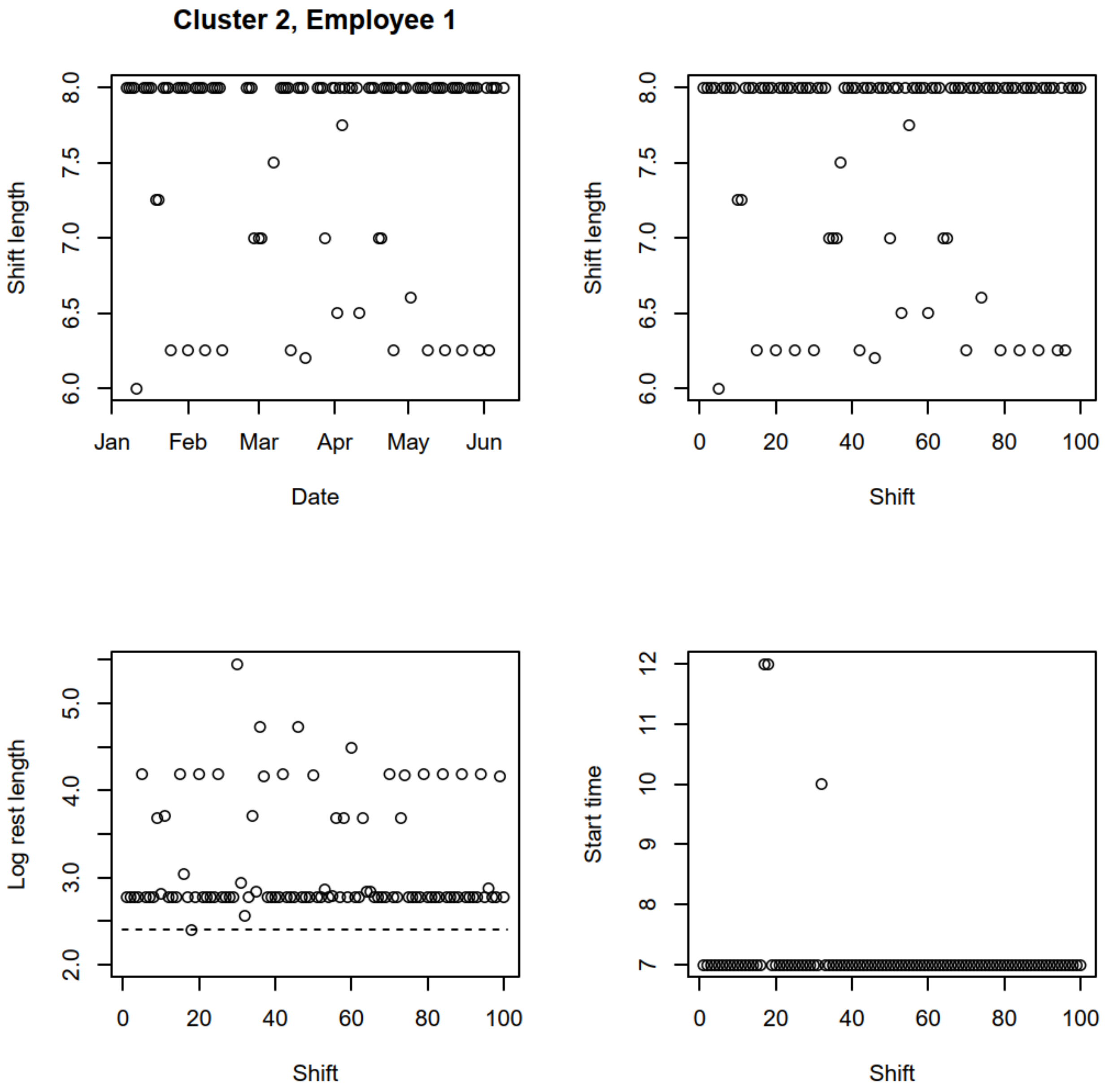
Cluster 2, Employee 1
Shift length
8.0
7.5
7.0
6.5
6.0
Jan
Feb
Mar
Apr
May
Jun
Date
Shift length
0
20
40
60
80
100
Shift
Log rest length
2.0
3.0
4.0
5.0
Shift
Start time
7
8
9
10
11
12
Shift

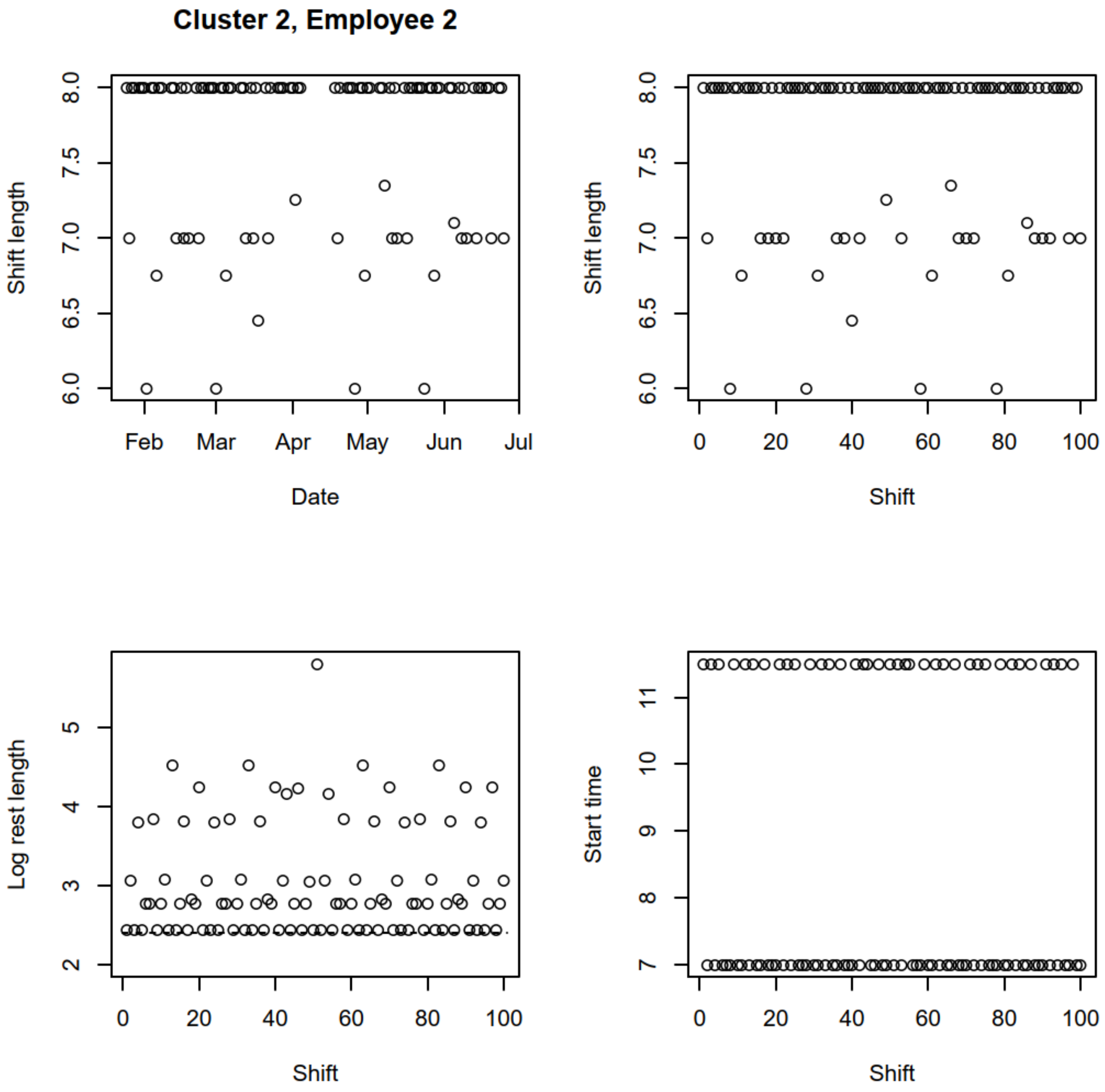
Cluster 2, Employee 2
Shift length
8.0
7.5
7.0
6.5
6.0
Feb
Mar
Apr
May
Jun
Jul
Date
Shift length
0
20
40
60
80
100
Shift
Log rest length
5
4
3
2
0
20
40
60
80
100
Shift
Start time
11
10
9
8
7
0
20
40
60
80
100
Shift

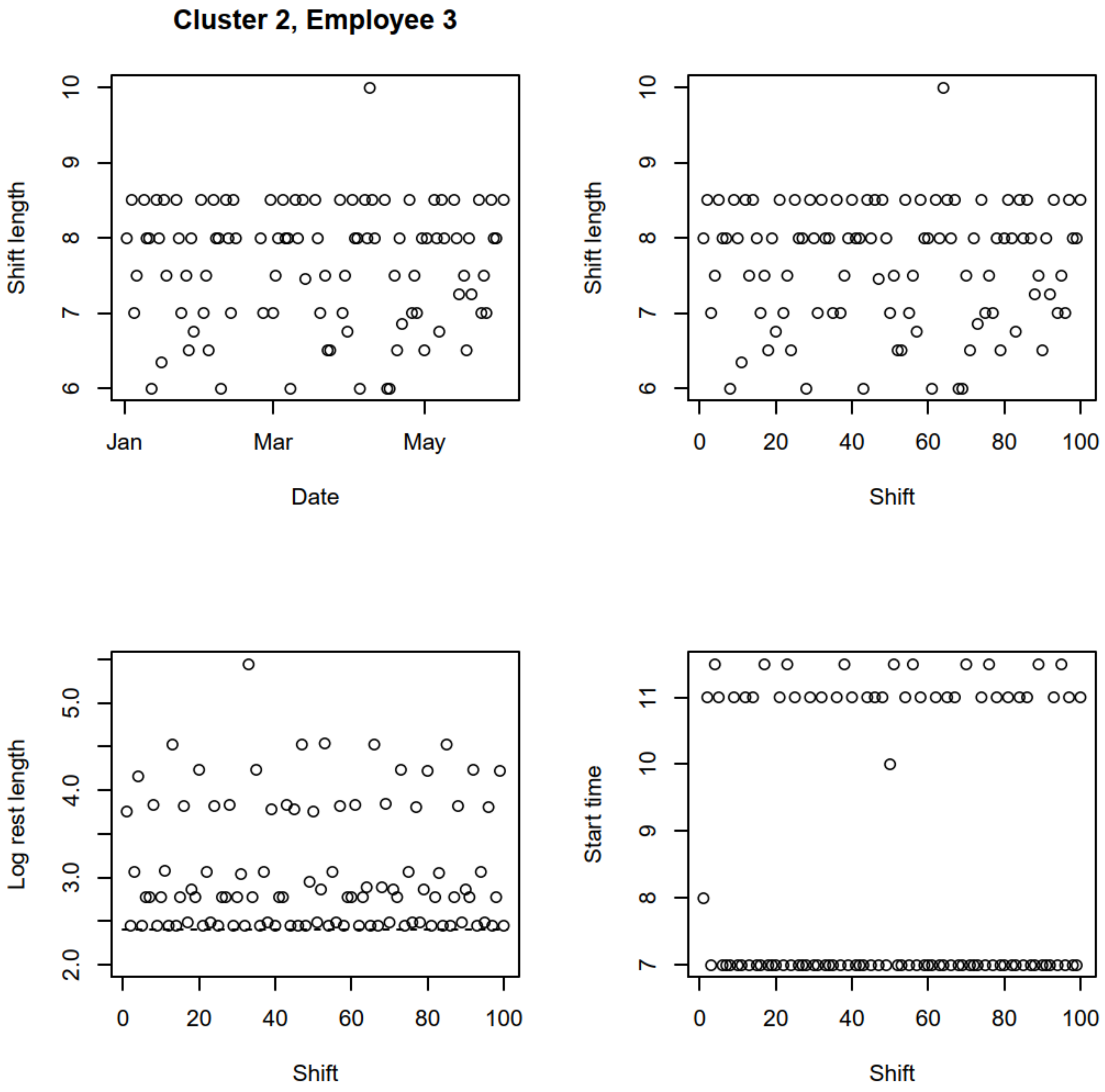

Cluster 2, Employee 3
Shift length
Date
Jan
Mar
May
Shift
Log rest length
Start time

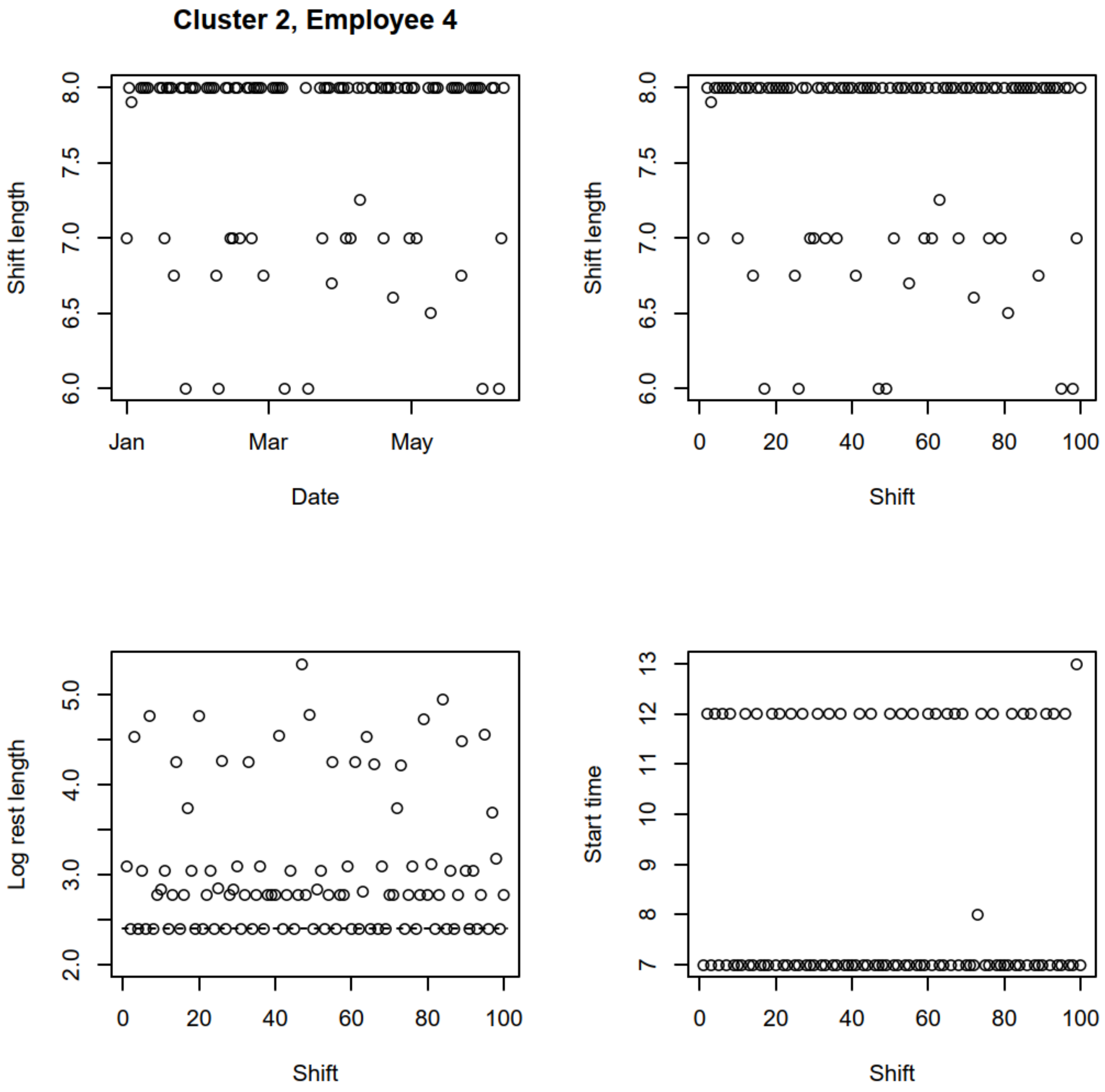
Cluster 2, Employee 4
Shift length
Date
Jan
Mar
May
Shift
Log rest length
Start time

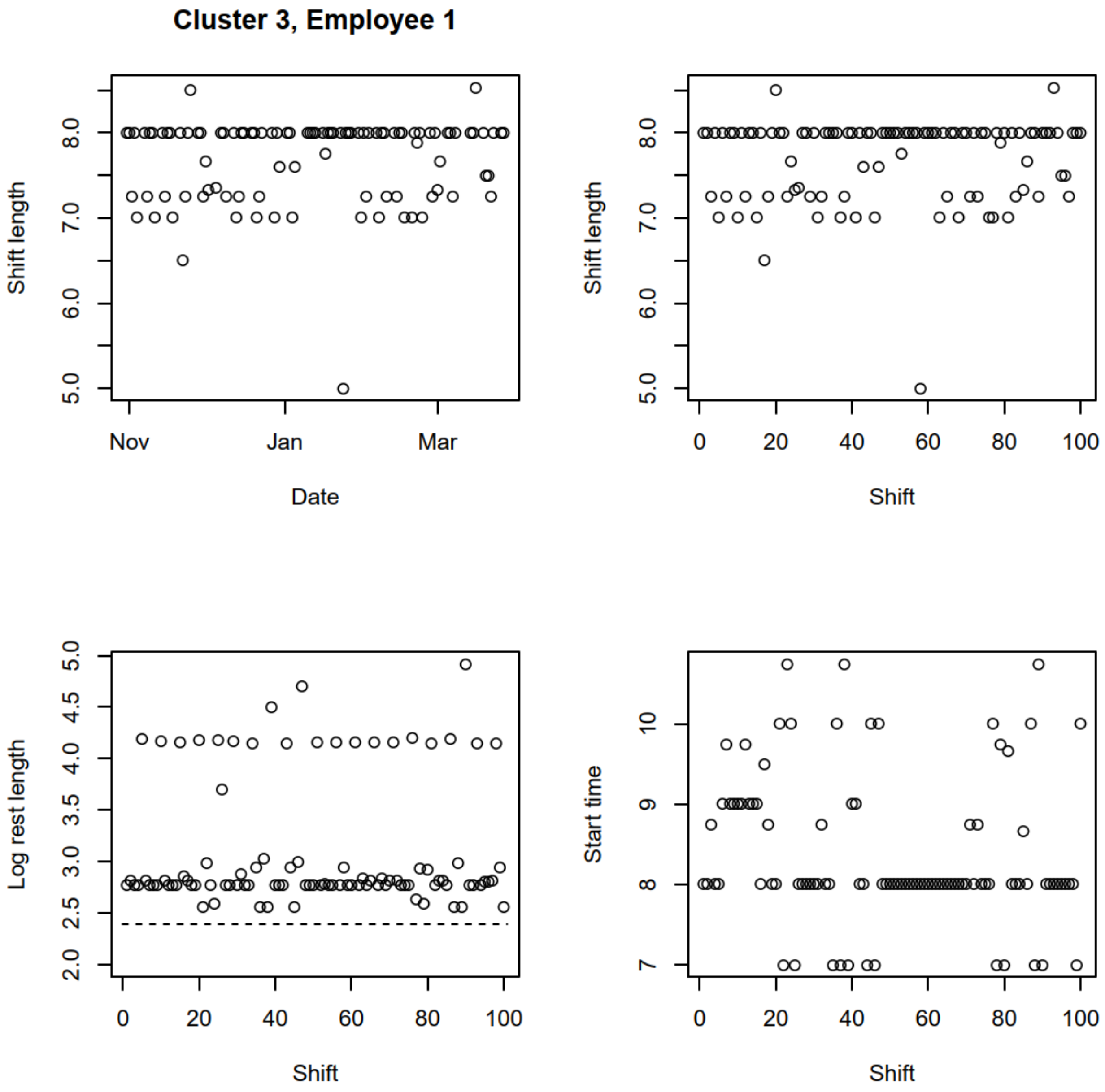
Cluster 3, Employee 1
Shift length
8.0
7.0
6.0
5.0
Nov
Jan
Mar
Date
Shift length
0
20
40
60
80
100
Shift
Log rest length
5.0
4.5
4.0
3.5
3.0
2.5
2.0
Shift
Start time
10
9
8
7
Shift

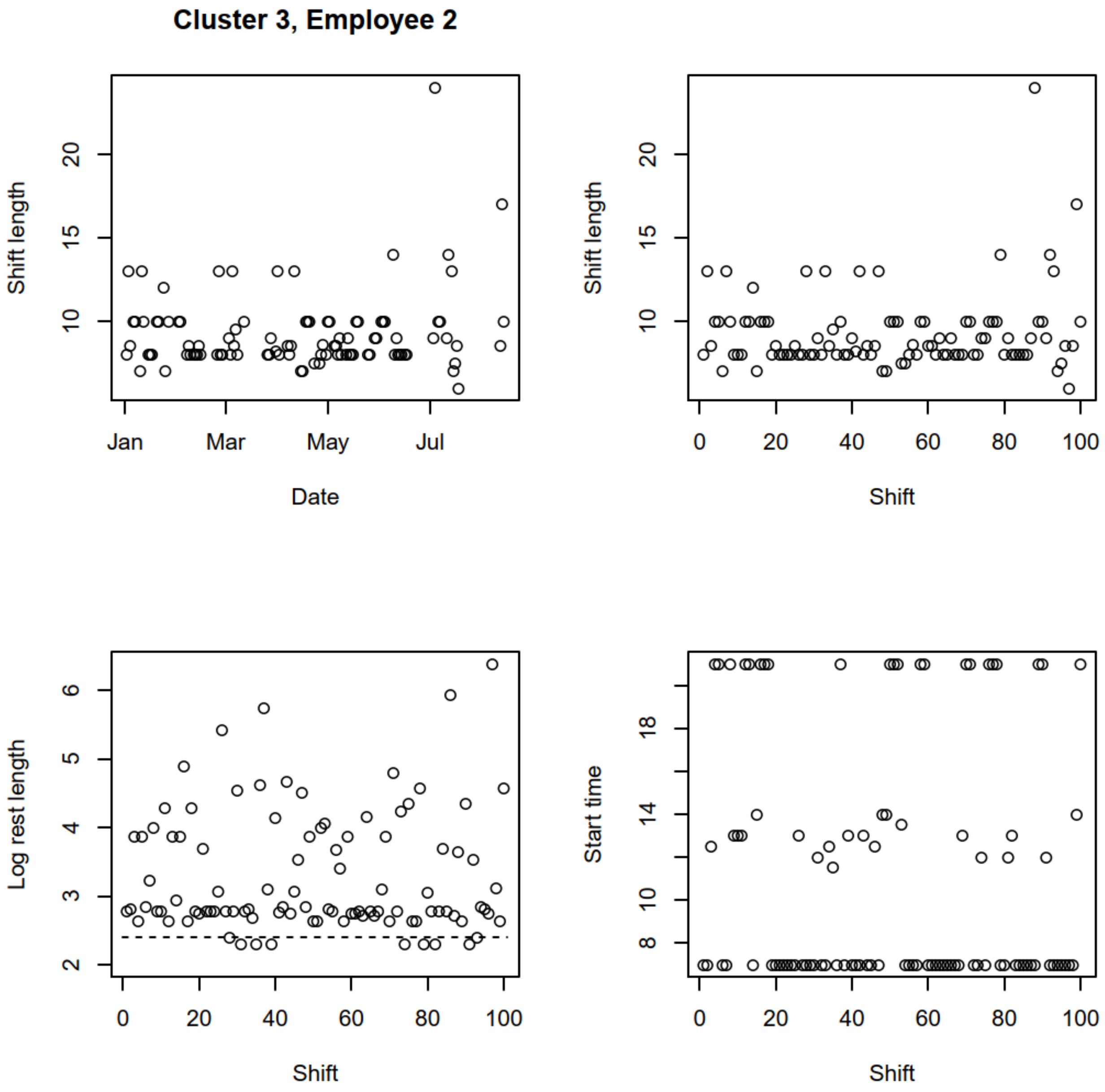
Cluster 3, Employee 2
Shift length
20
15
10
Jan
Mar
May
Jul
Date
Shift length
0
20
40
60
80
100
Shift
Log rest length
6
5
4
3
2
Shift
Start time
18
14
10
8
Shift

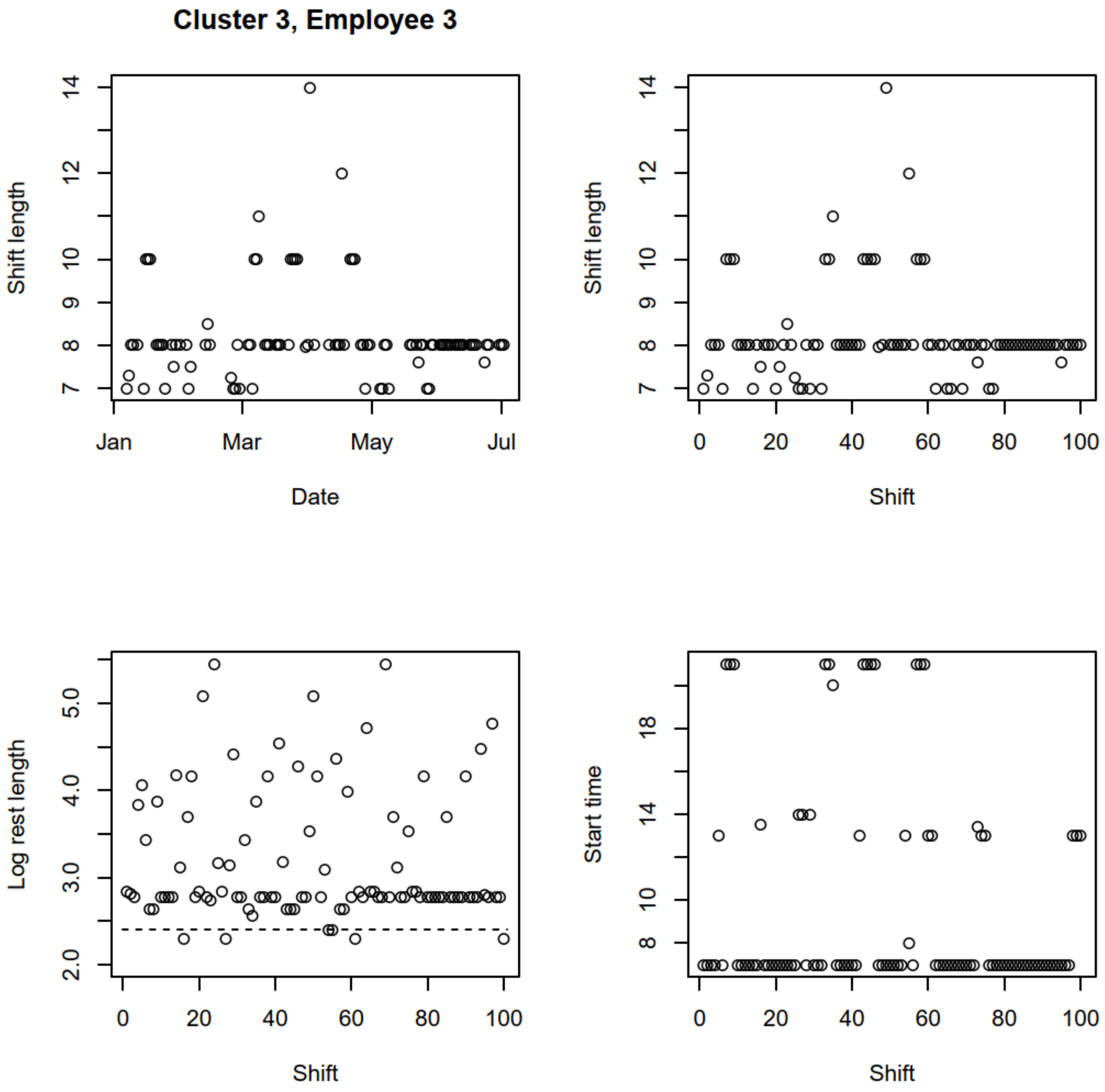
Cluster 3, Employee 3
Shift length
Date
Jan
Mar
May
Jul
Shift
Log rest length
Start time

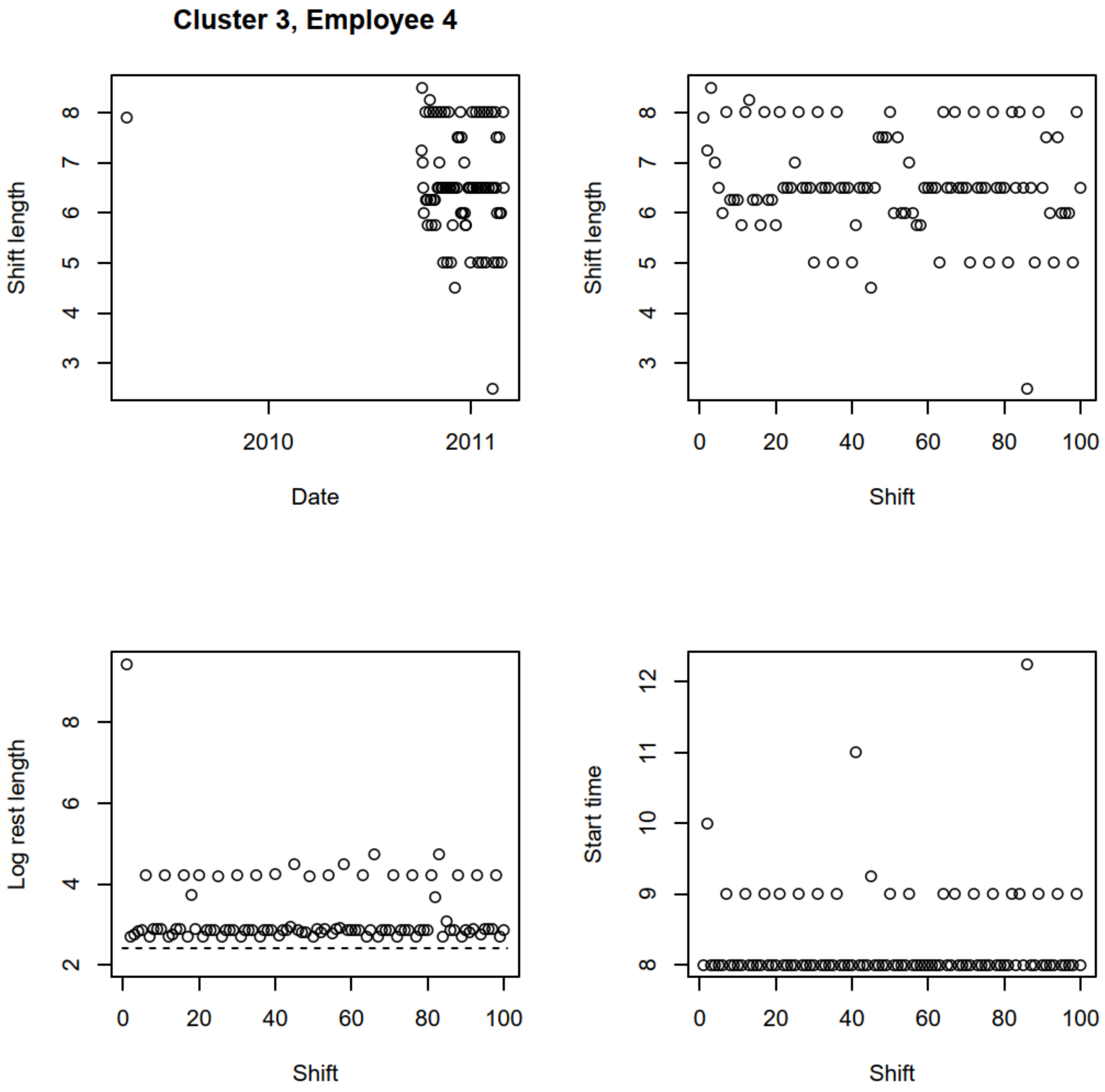
Cluster 3, Employee 4
Shift length
Date
2010
2011
Shift length
Shift
Log rest length
Shift
Start time
Shift

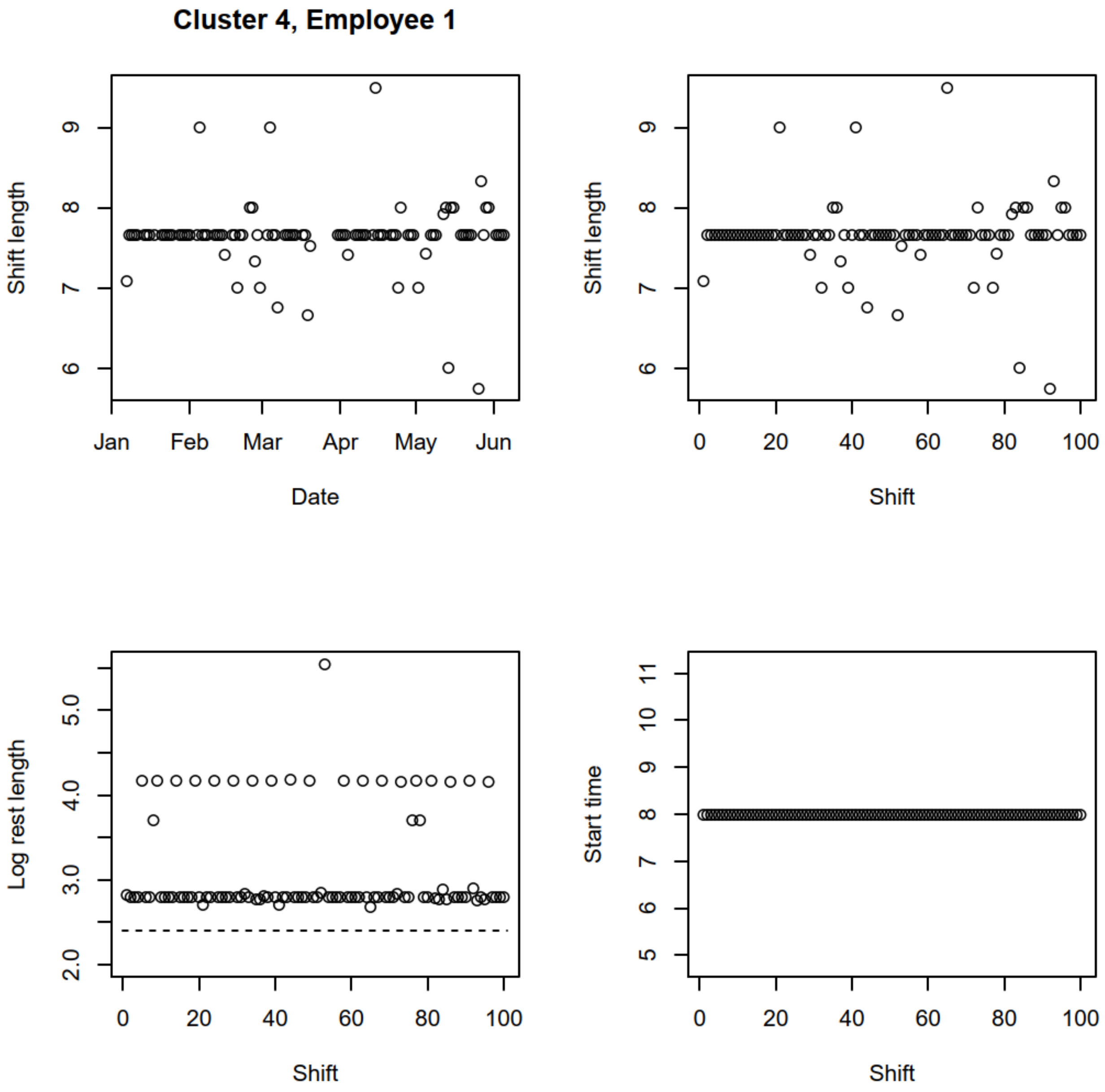
Cluster 4, Employee 1
Shift length
Date
Jan
Feb
Mar
Apr
May
Jun
Shift
Log rest length
Start time

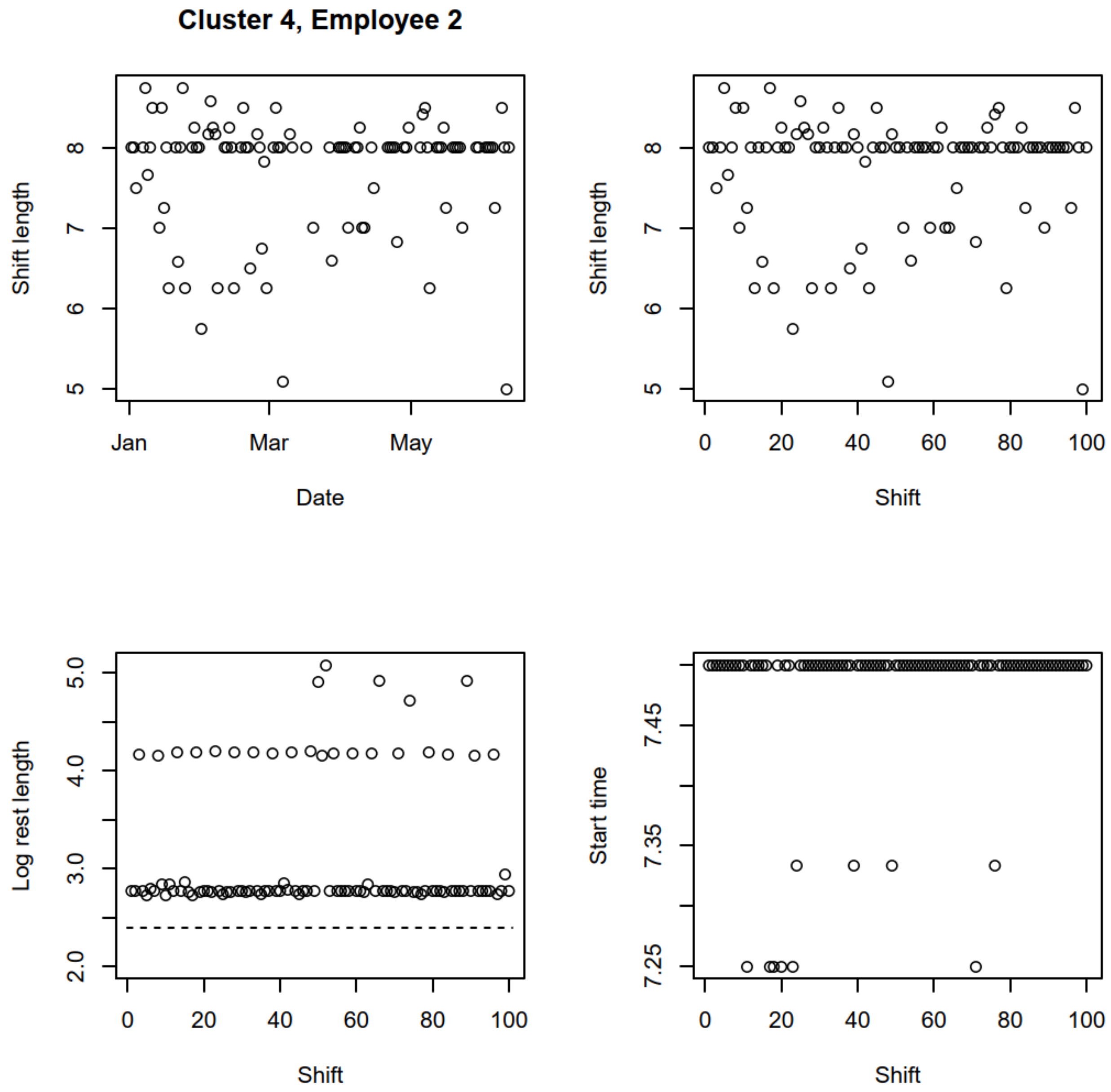
Cluster 4, Employee 2
Shift length
Date
Jan
Mar
May
Shift
Log rest length
Start time

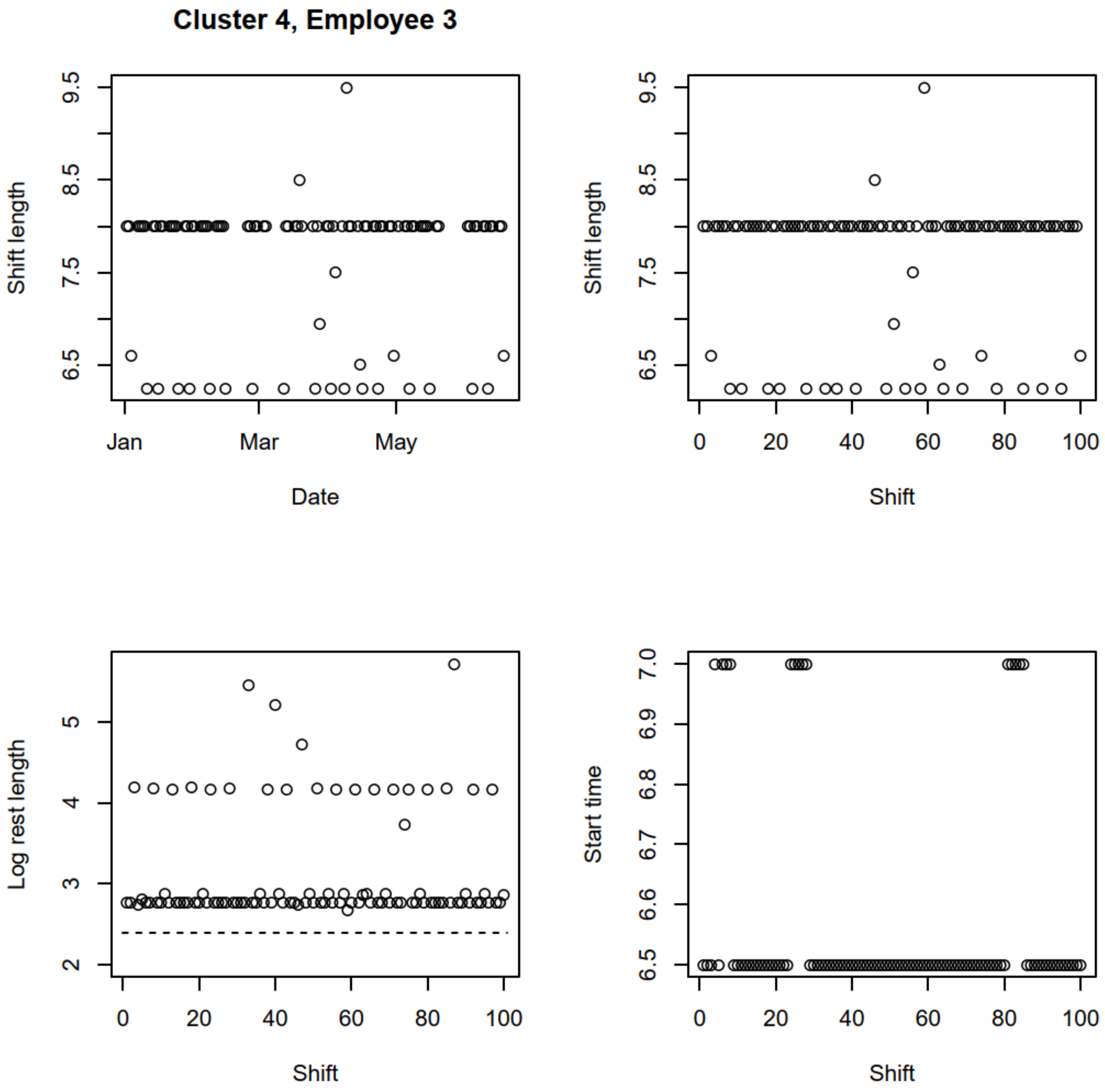
Cluster 4, Employee 3
Shift length
9.5
8.5
7.5
6.5
Jan
Mar
May
Date
Shift length
9.5
8.5
7.5
6.5
0
20
40
60
80
100
Shift
Log rest length
5
4
3
2
0
20
40
60
80
100
Shift
Start time
7.0
6.9
6.8
6.7
6.6
6.5
0
20
40
60
80
100
Shift

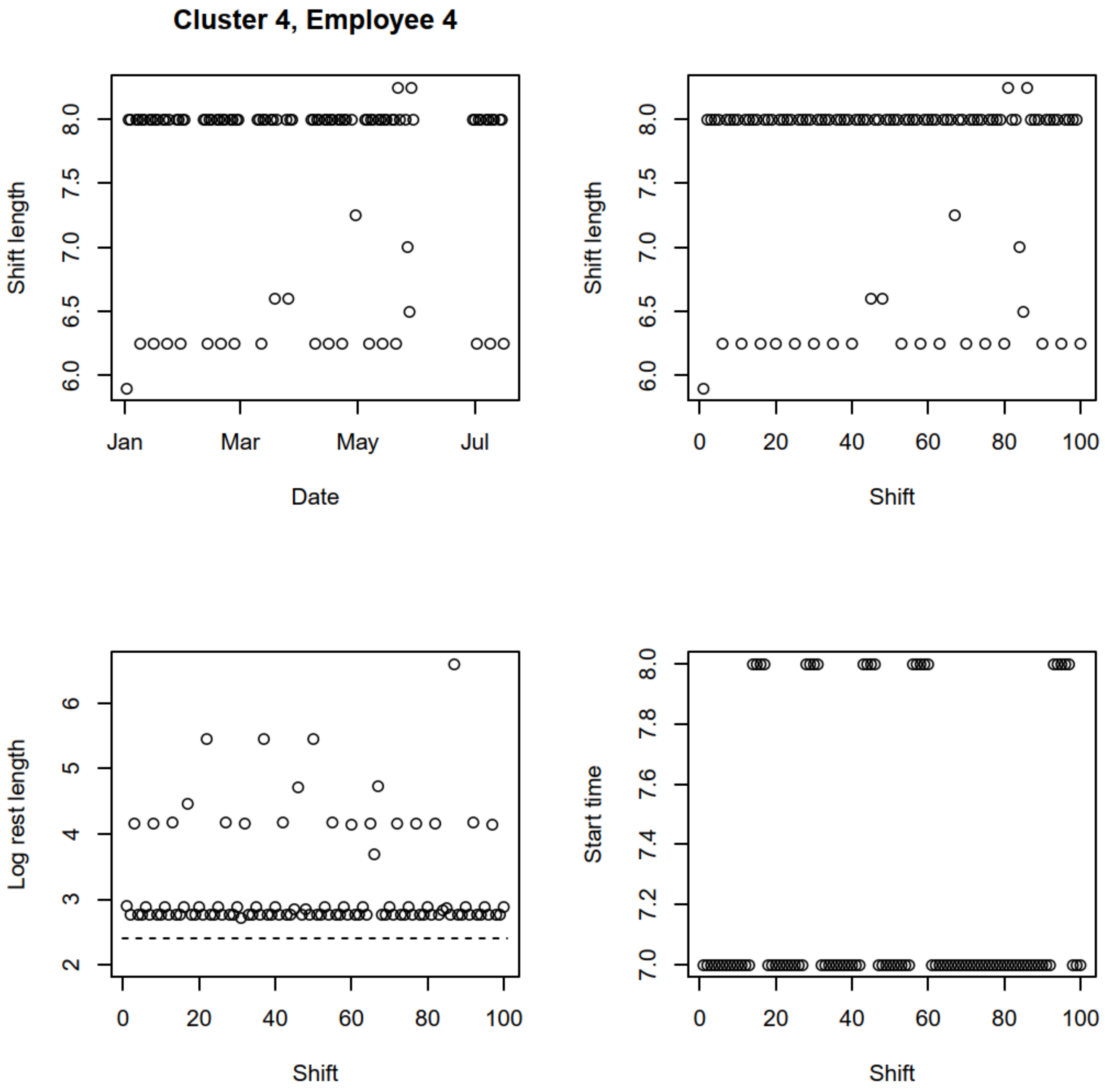
Cluster 4, Employee 4
Shift length
Date
Jan
Mar
May
Jul
Shift
Log rest length
Start time

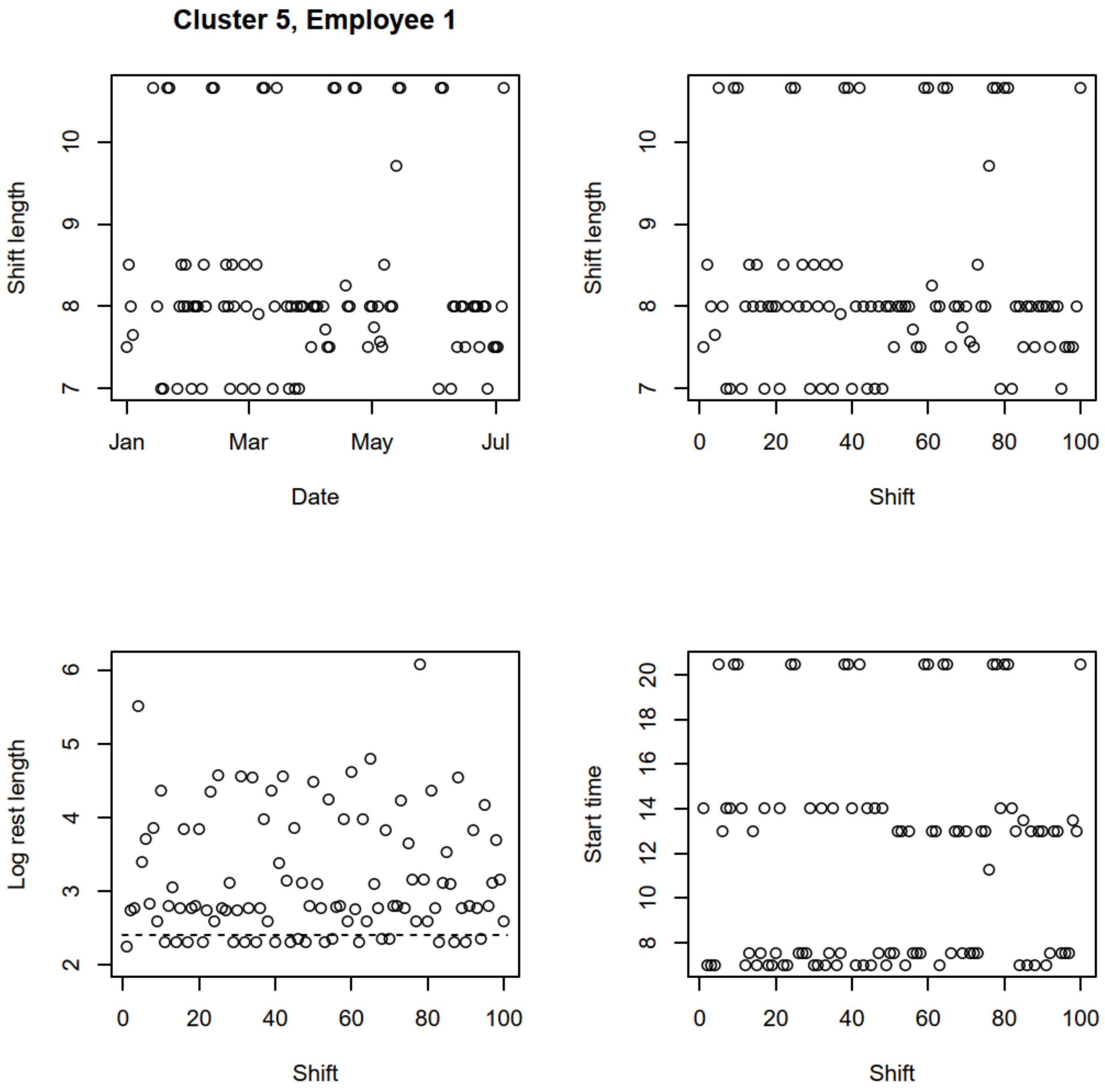

Cluster 5, Employee 1
Shift length
10
9
8
7
Jan
Mar
May
Jul
Date
Shift length
10
9
8
7
0
20
40
60
80
100
Shift
Log rest length
6
5
4
3
2
0
20
40
60
80
100
Shift
Start time
20
18
16
14
12
10
8
0
20
40
60
80
100
Shift

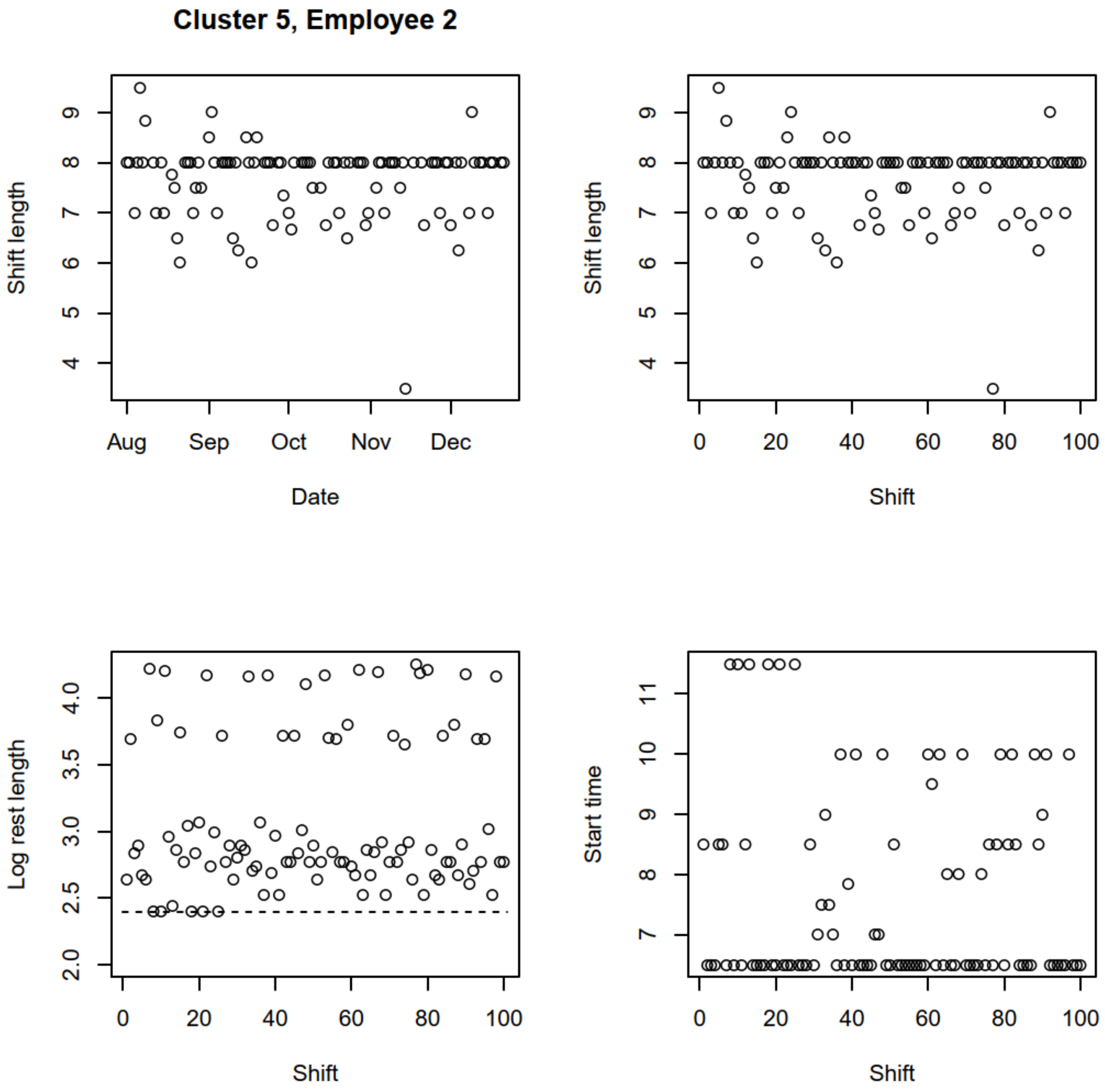
Cluster 5, Employee 2
Shift length
Date
Aug
Sep
Oct
Nov
Dec
Shift
Log rest length
Start time

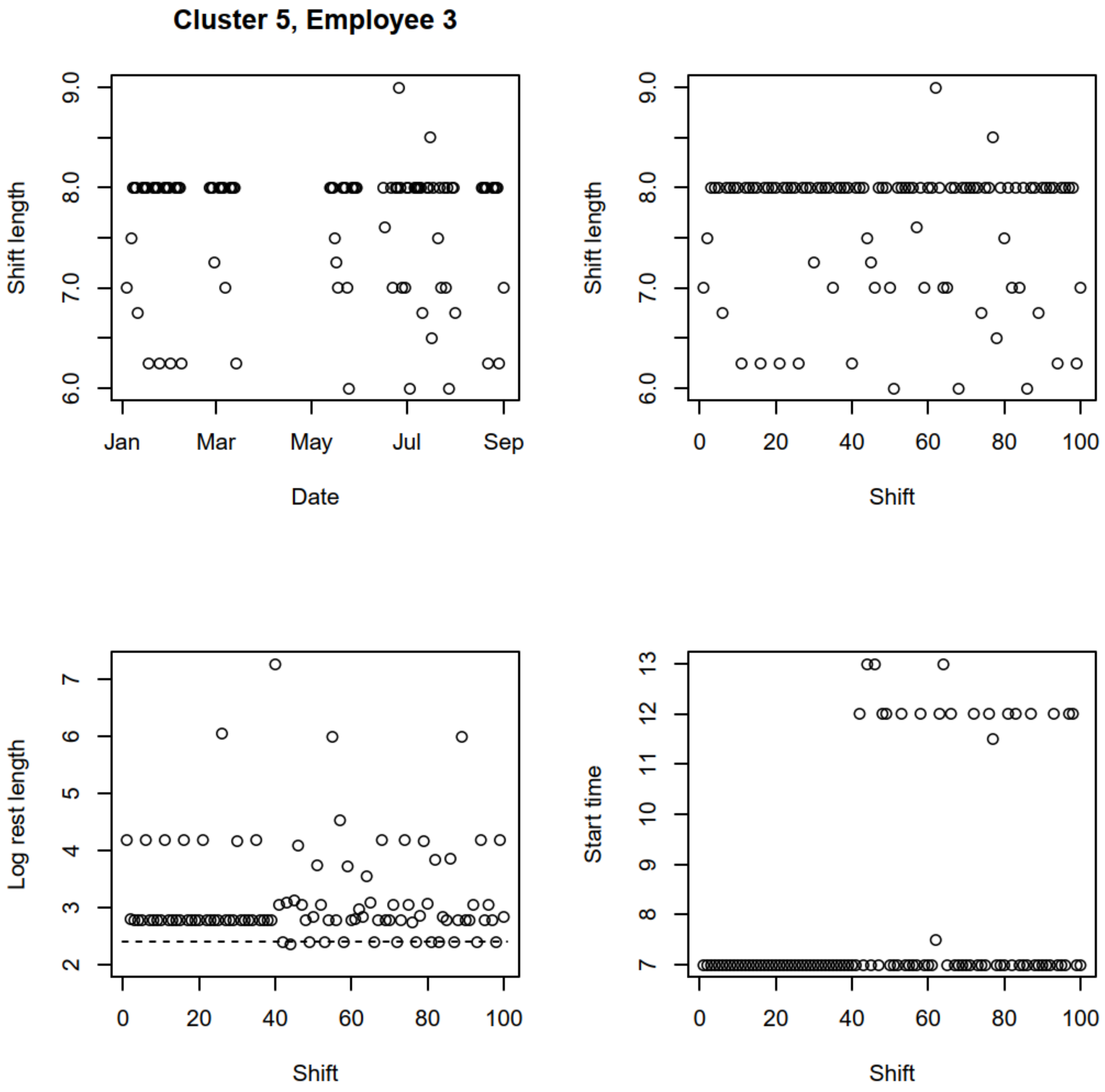
Cluster 5, Employee 3
Shift length
9.0
8.0
7.0
6.0
Jan
Mar
May
Jul
Sep
Date
Shift length
0
20
40
60
80
100
Shift
Log rest length
7
6
5
4
3
2
Shift
Start time
13
12
11
10
9
8
7
Shift

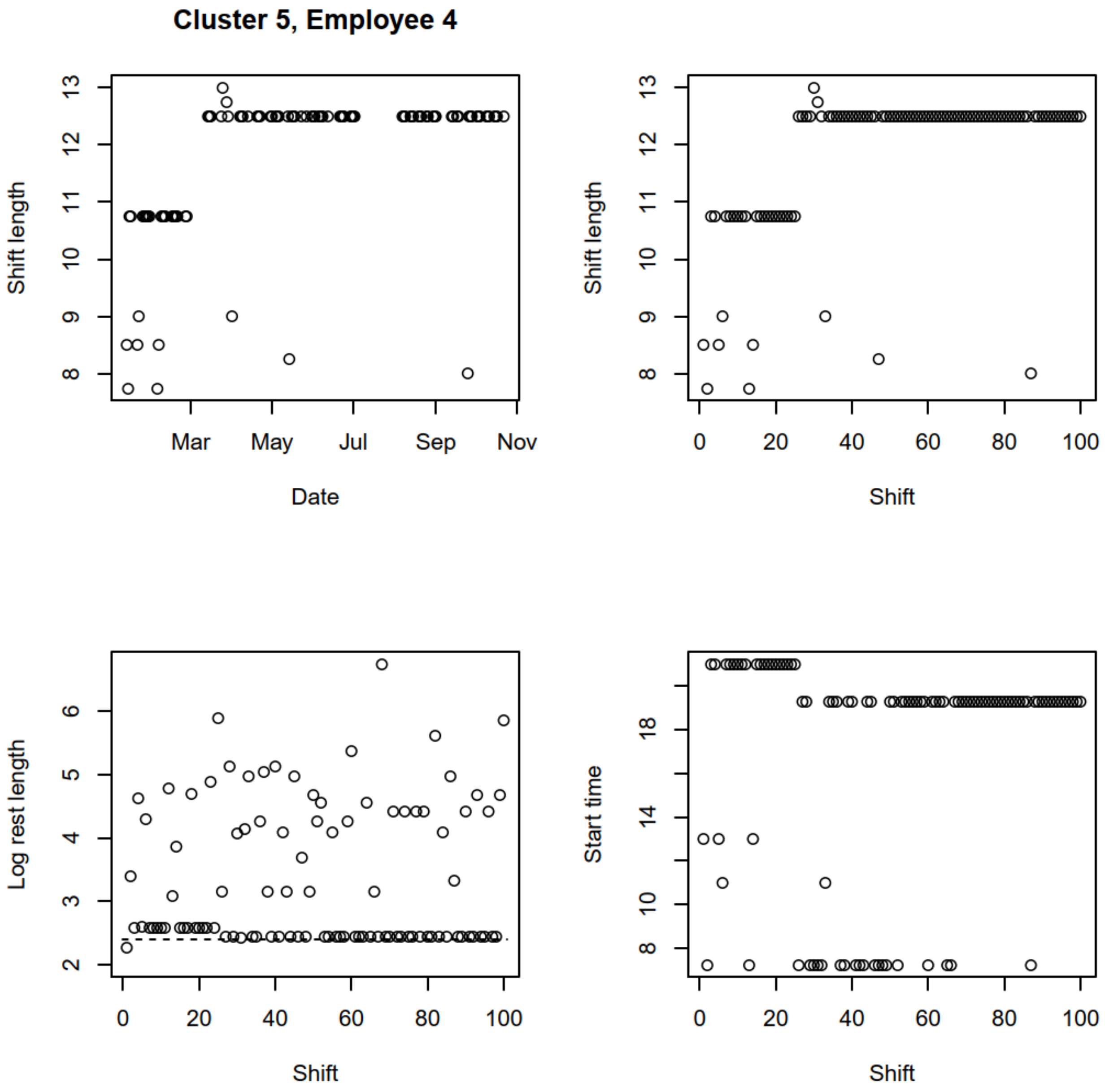
Cluster 5, Employee 4
Shift length
Date
Mar
May
Jul
Sep
Nov
Shift
Log rest length
Start time

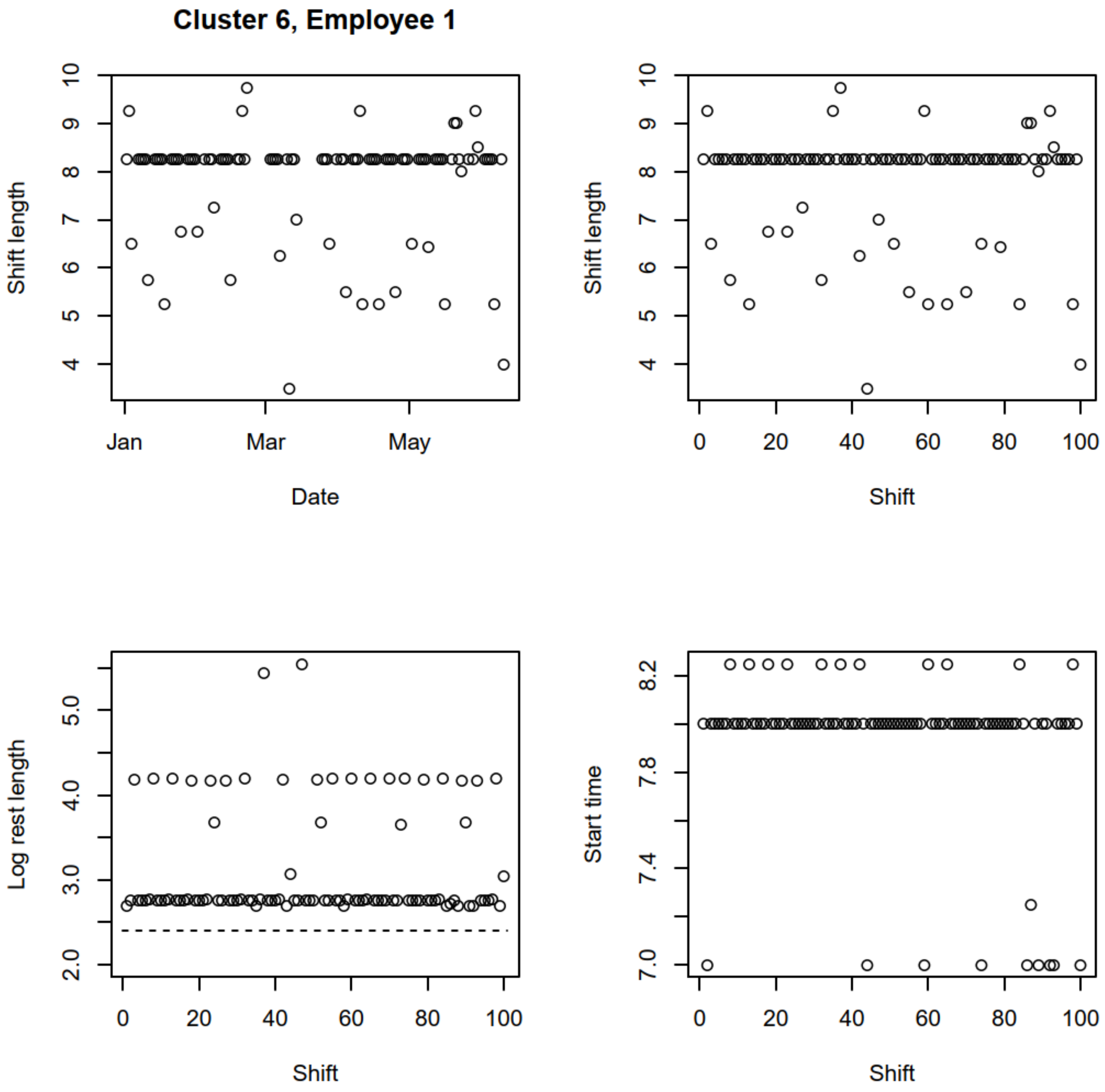
Cluster 6, Employee 1
Shift length
4
5
6
7
8
9
10
Jan
Mar
May
Date
Shift length
0
20
40
60
80
100
Shift
Log rest length
2.0
3.0
4.0
5.0
Shift
Start time
7.0
7.4
7.8
8.2
Shift

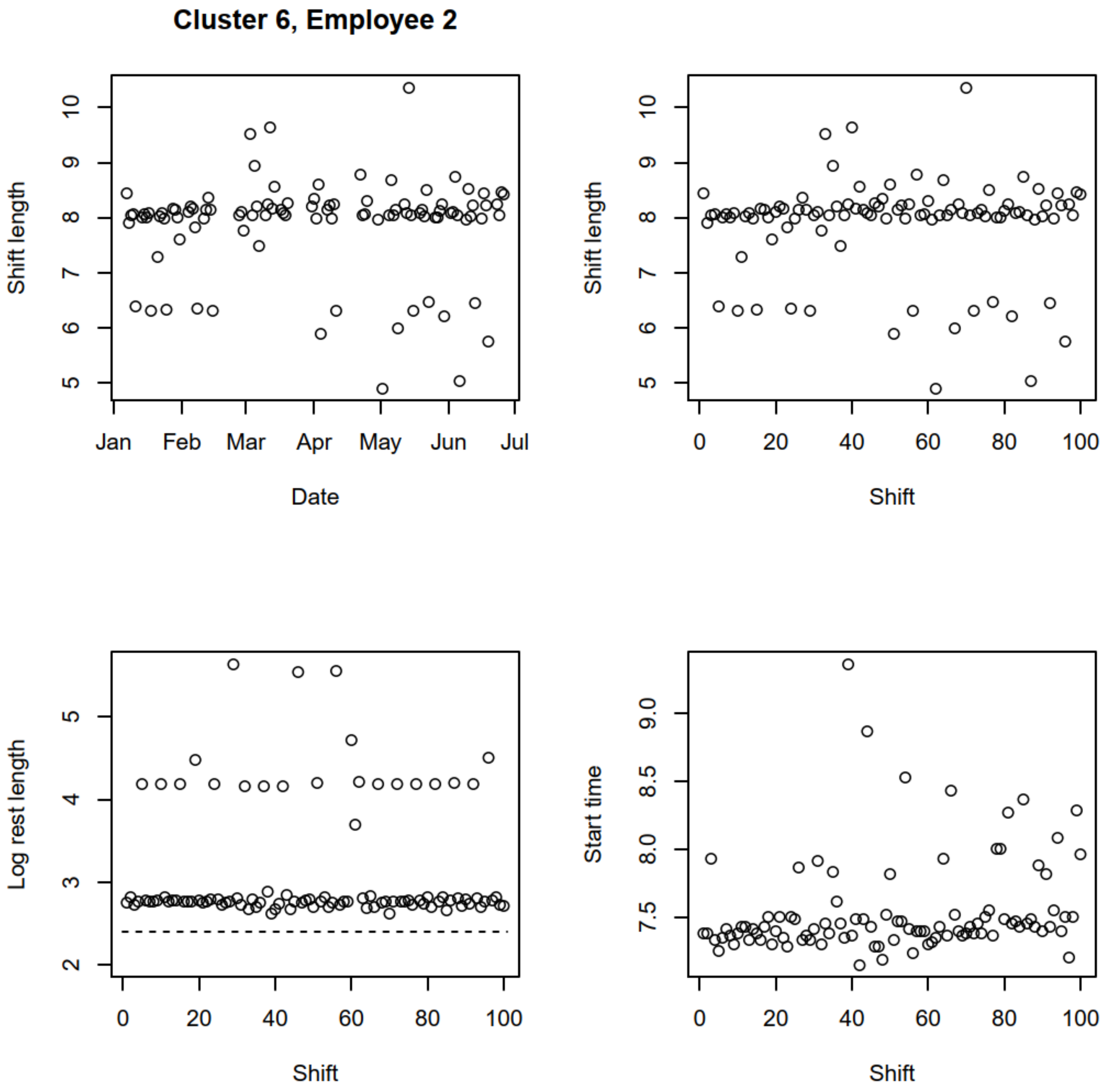
Cluster 6, Employee 2
Shift length
Date
Jan
Feb
Mar
Apr
May
Jun
Jul
Shift
Log rest length
Start time

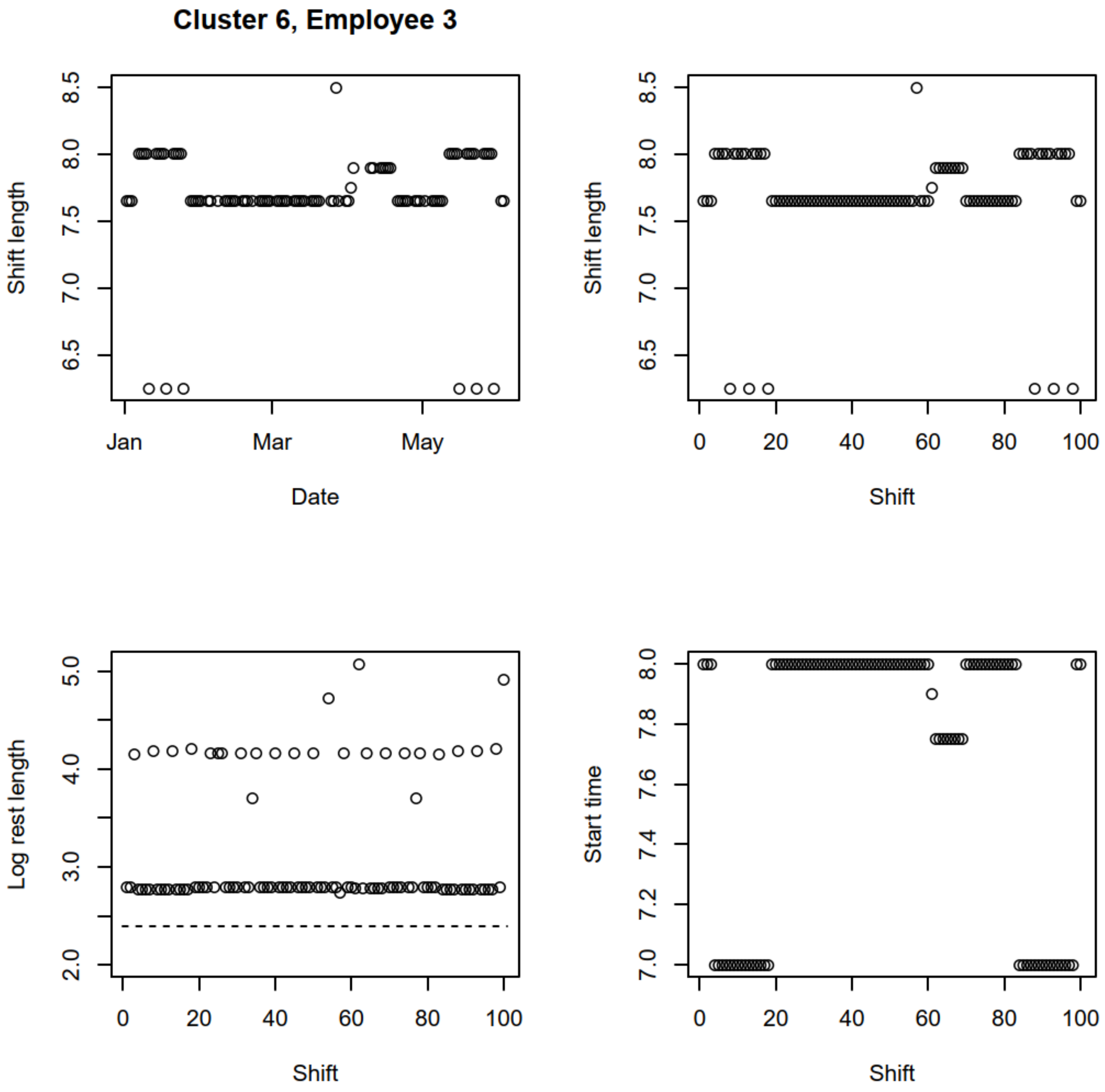
Cluster 6, Employee 3
Shift length
8.5
8.0
7.5
7.0
6.5
Jan
Mar
May
Date
Shift length
0
20
40
60
80
100
Shift
Log rest length
5.0
4.0
3.0
2.0
Shift
Start time
8.0
7.8
7.6
7.4
7.2
7.0
Shift

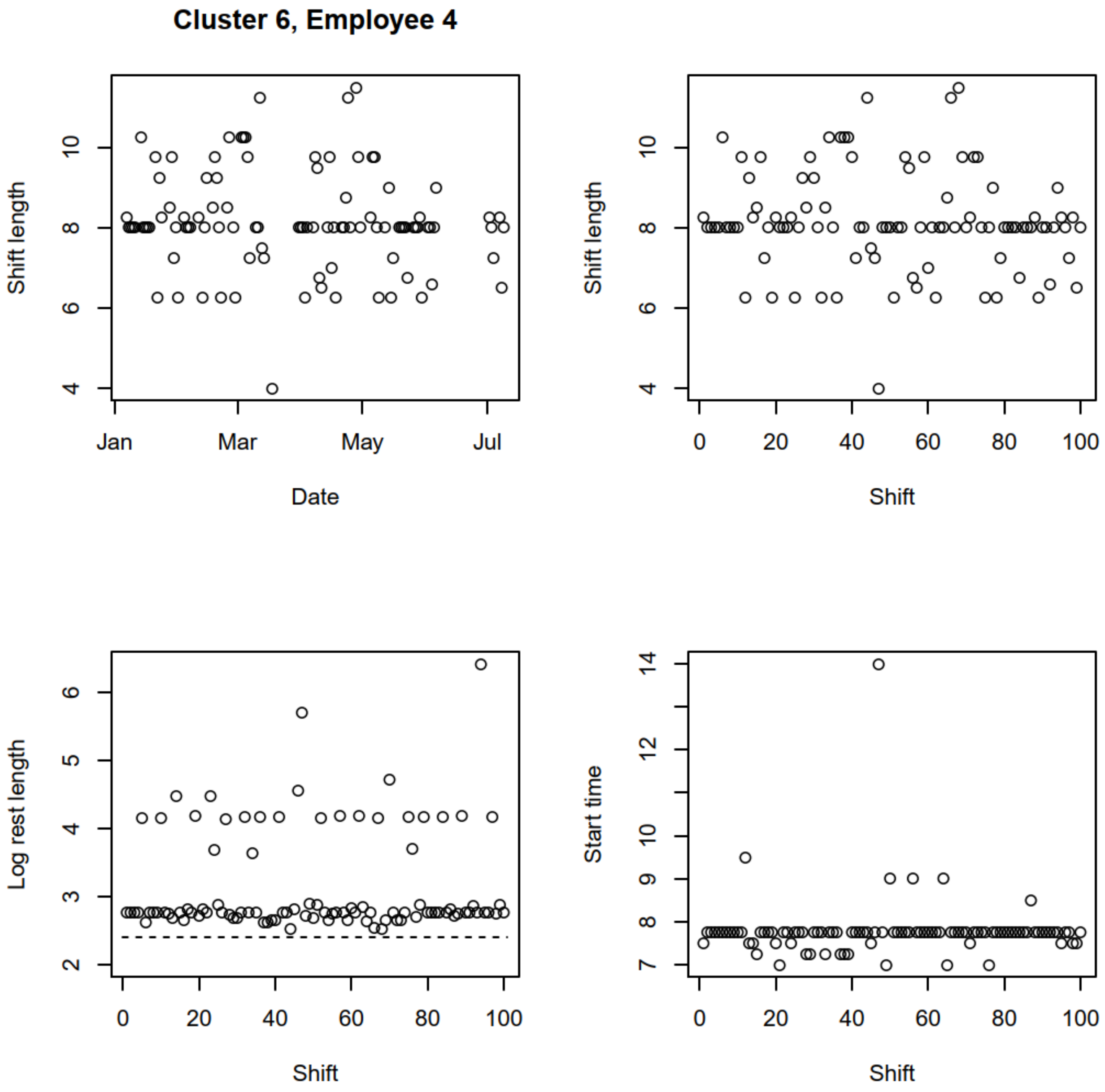

Cluster 6, Employee 4
Shift length
Date
Jan
Mar
May
Jul
Shift
Log rest length
Start time

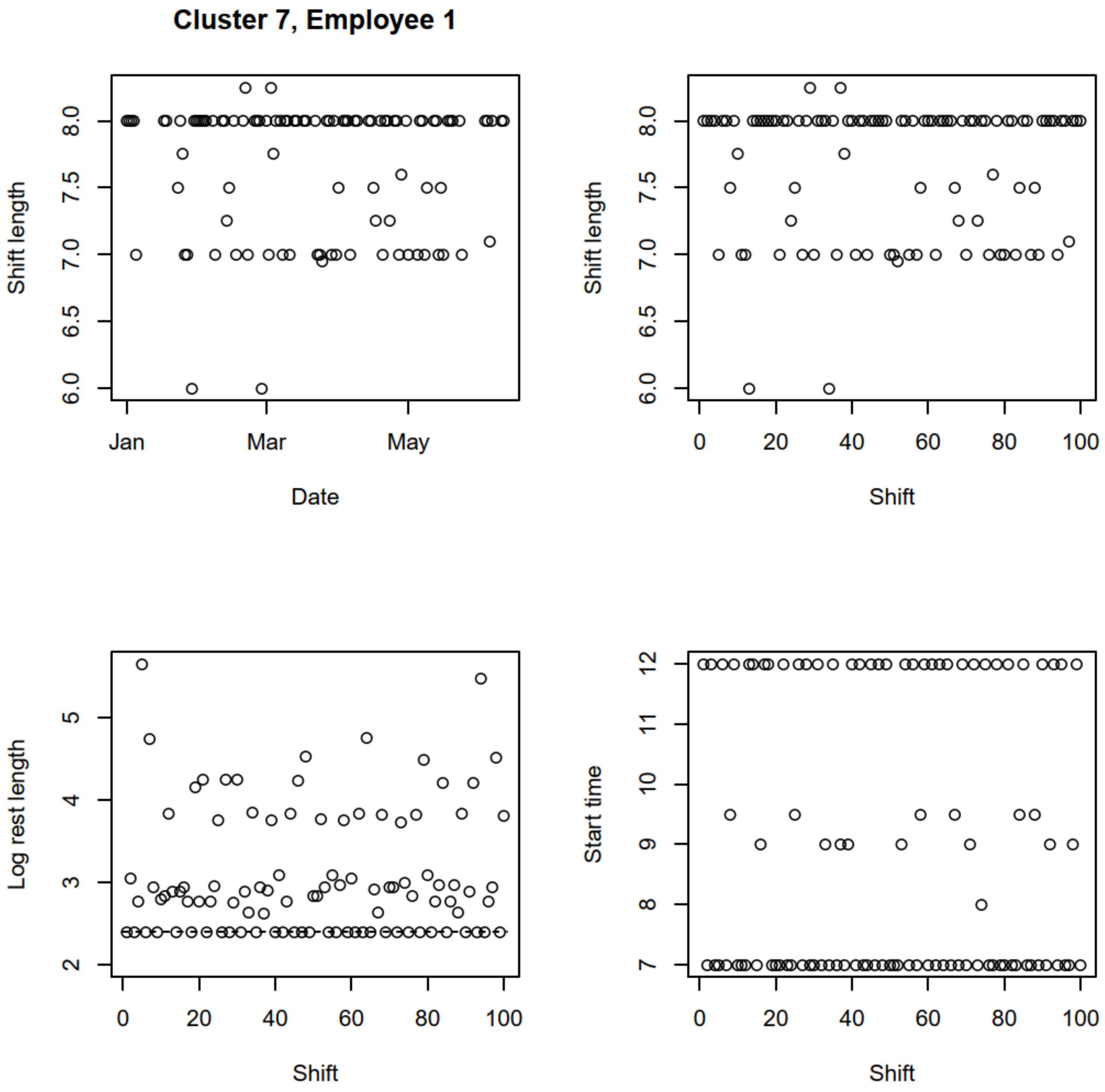
Cluster 7, Employee 1
Shift length
8.0
7.5
7.0
6.5
6.0
Jan
Mar
May
Date
Shift length
0
20
40
60
80
100
Shift
Log rest length
5
4
3
2
Shift
Start time
12
11
10
9
8
7
Shift

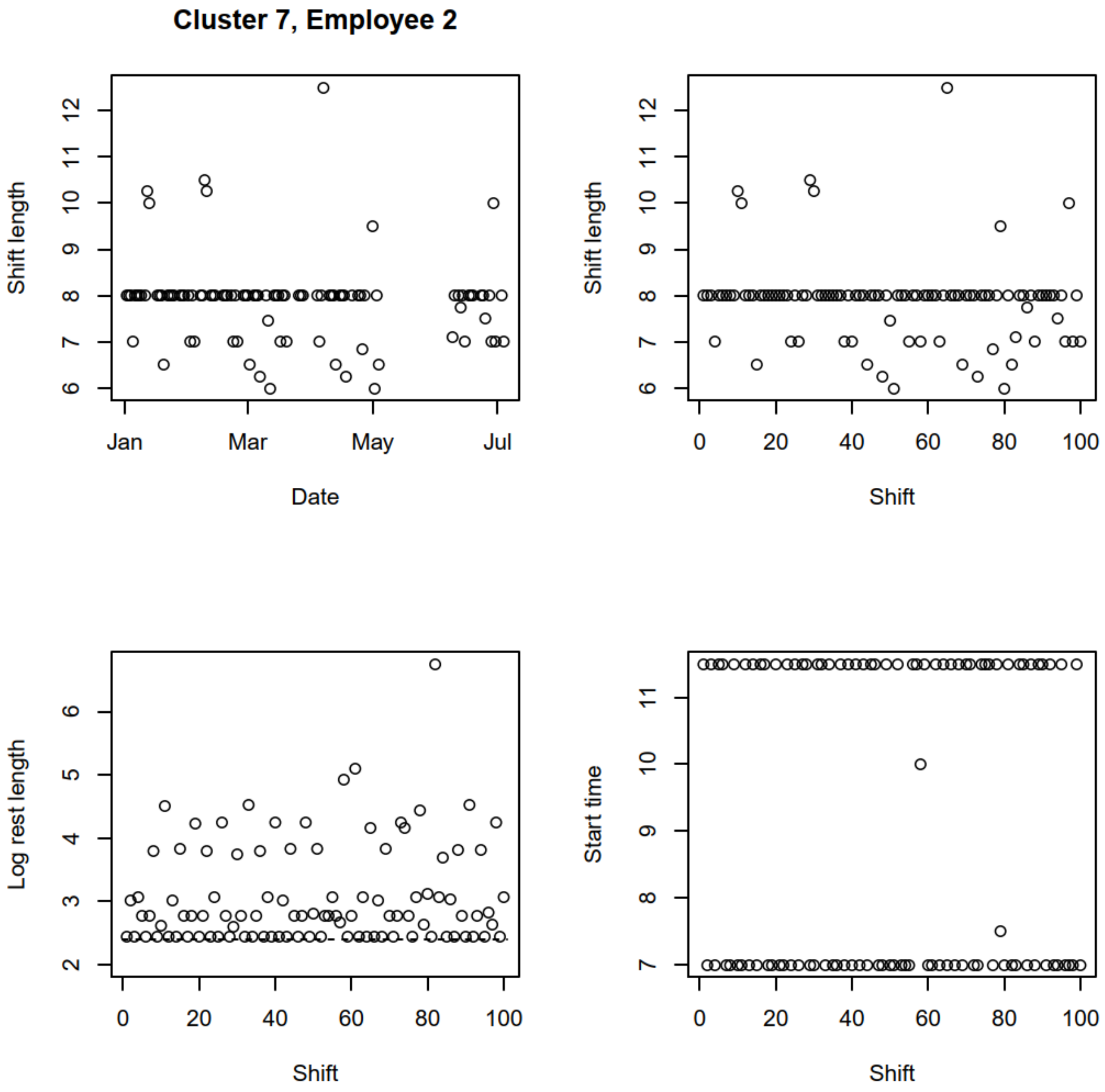
Cluster 7, Employee 2
Shift length
Date
Jan
Mar
May
Jul
Shift length
Shift
Log rest length
Shift
Start time
Shift

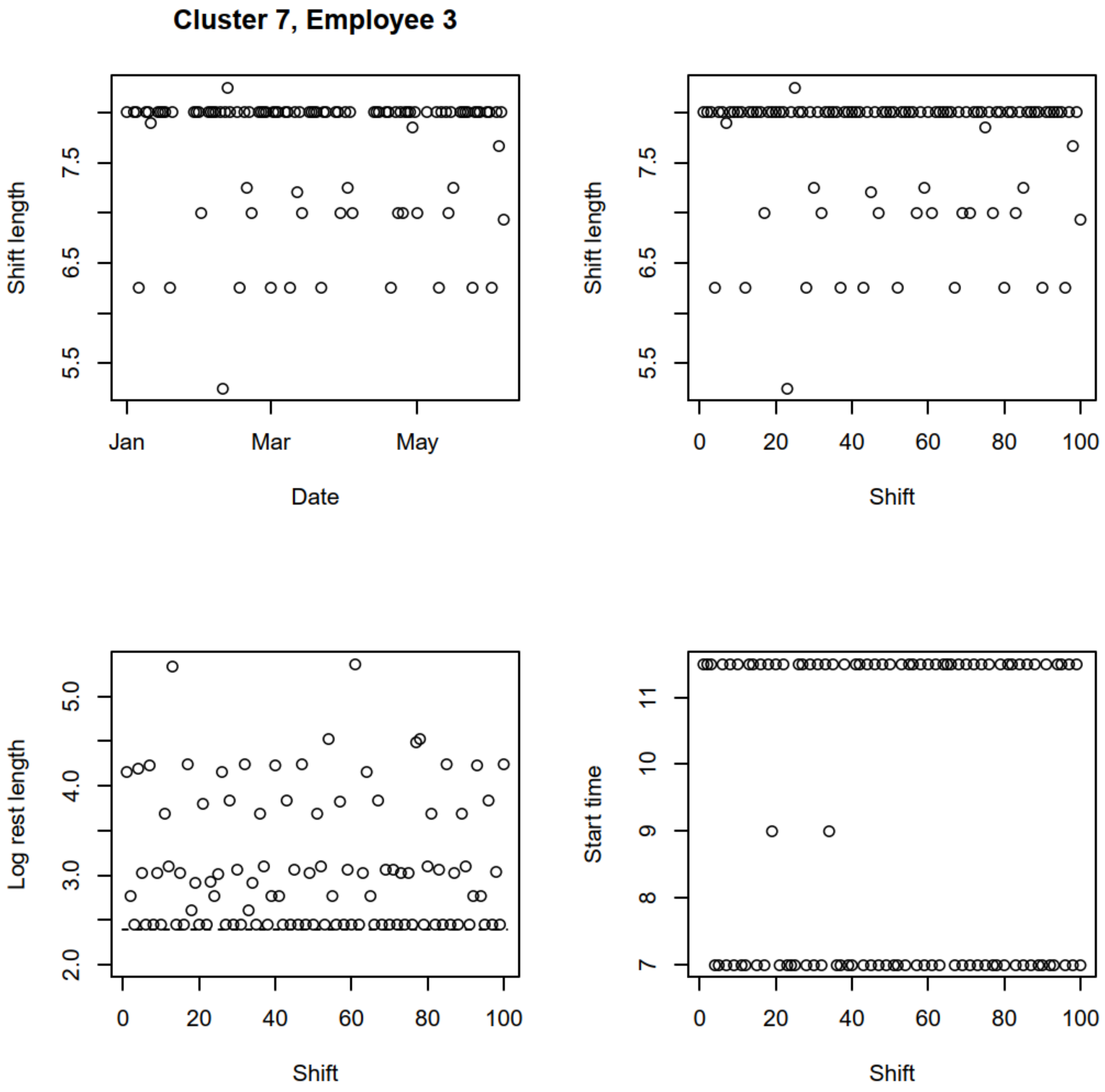
Cluster 7, Employee 3
Shift length
5.5
6.5
7.5
Jan
Mar
May
Date
Shift length
5.5
6.5
7.5
0
20
40
60
80
100
Shift
Log rest length
2.0
3.0
4.0
5.0
0
20
40
60
80
100
Shift
Start time
7
8
9
10
11
0
20
40
60
80
100
Shift

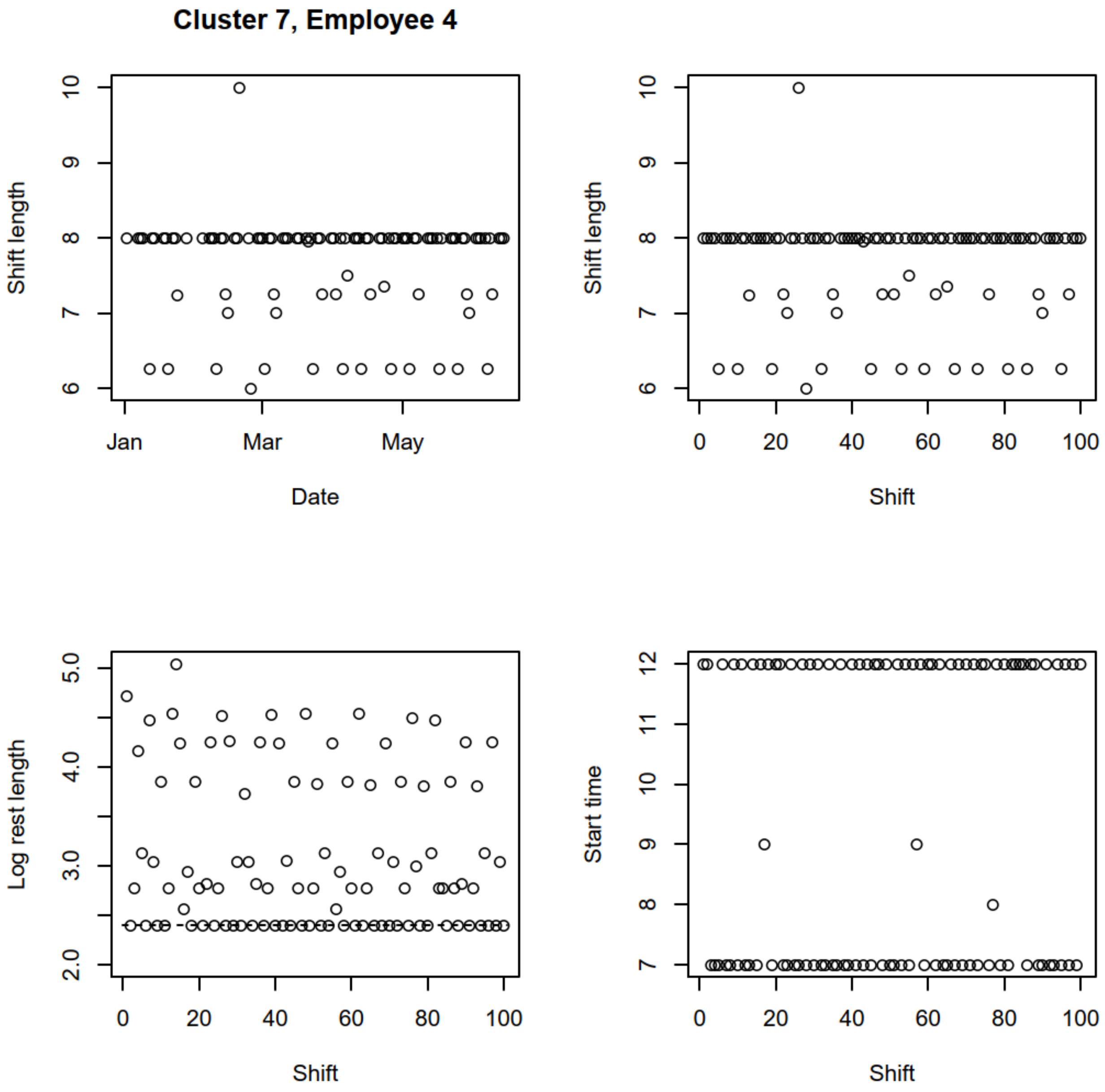
Cluster 7, Employee 4
Shift length
Date
Jan
Mar
May
Shift length
Shift
Log rest length
Shift
Start time
Shift

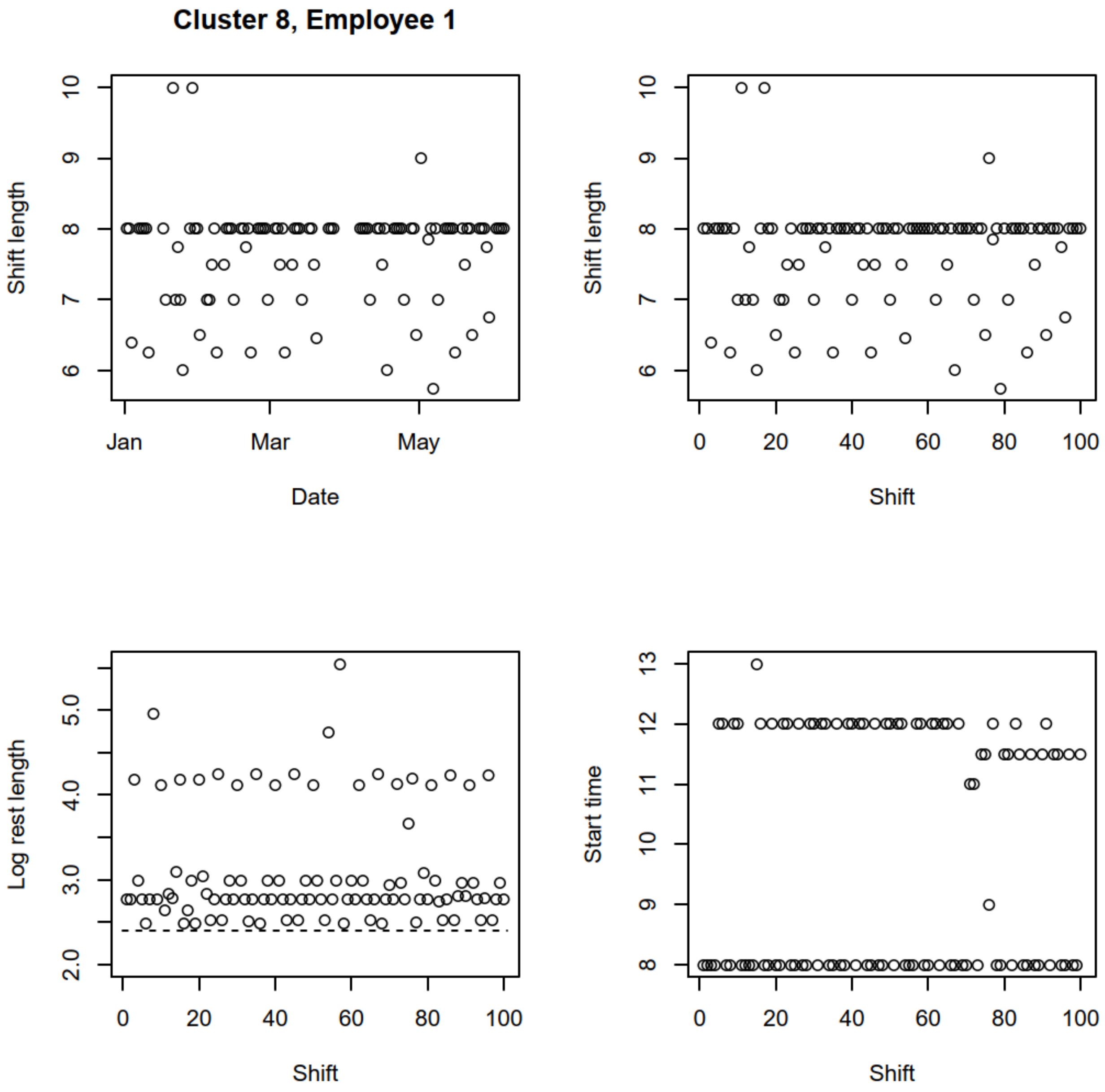
Cluster 8, Employee 1
Shift length
Date
Jan
Mar
May
Shift length
Shift
Log rest length
Shift
Start time
Shift

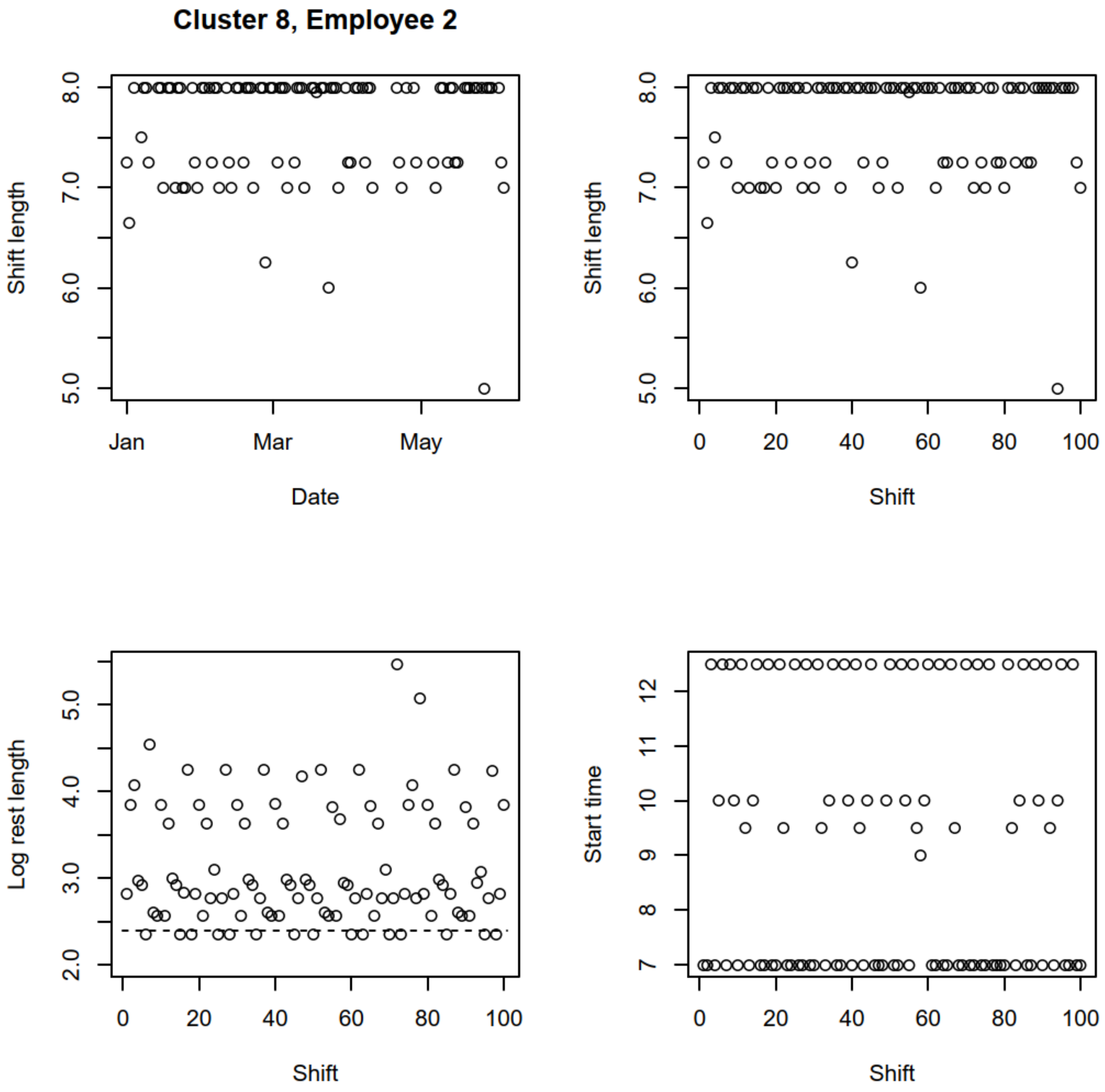
Cluster 8, Employee 2
Shift length
Date
Jan
Mar
May
Shift
Log rest length
Start time

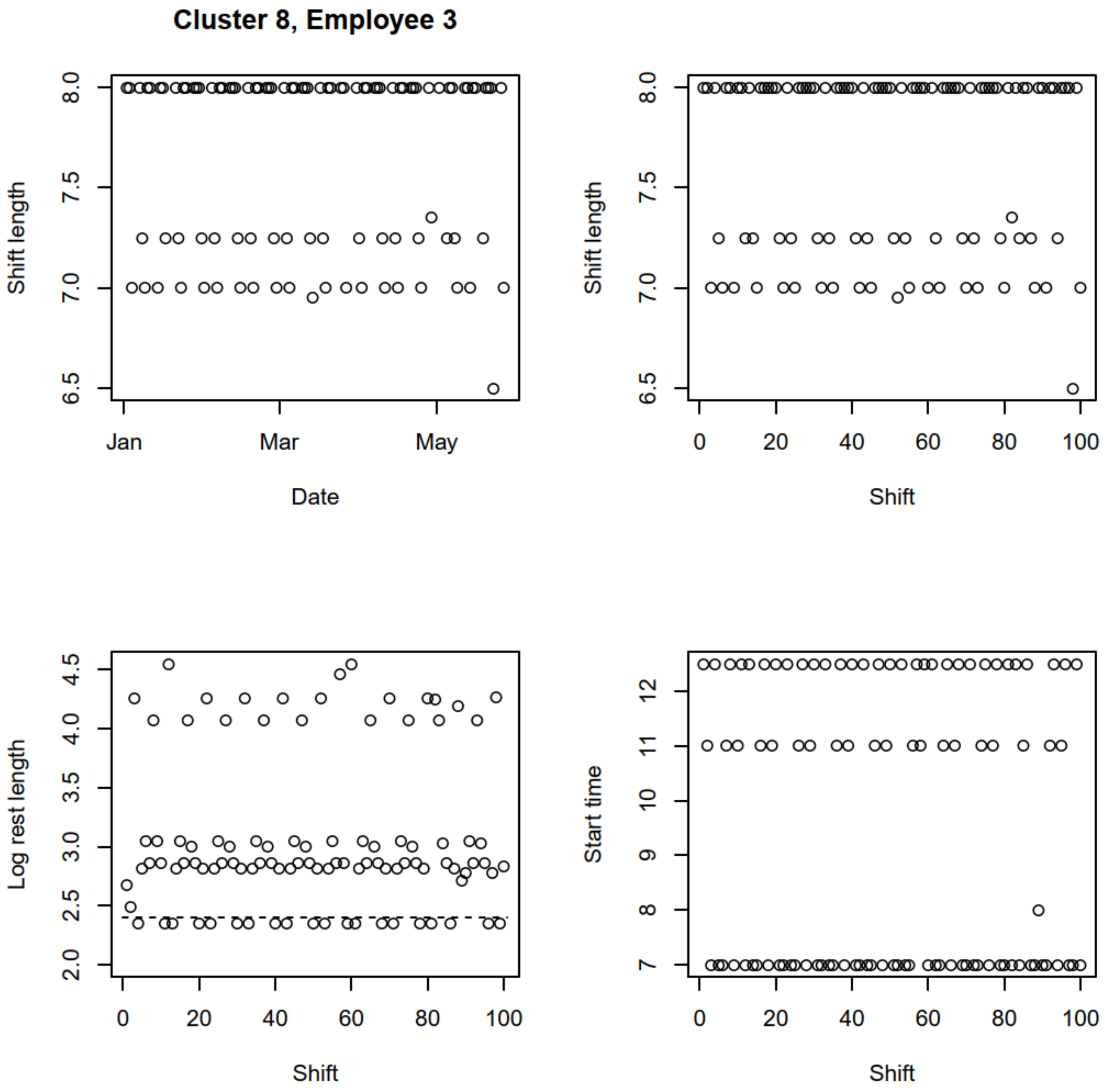
Cluster 8, Employee 3
Shift length
6.5
7.0
7.5
8.0
Jan
Mar
May
Date
Shift length
6.5
7.0
7.5
8.0
0
20
40
60
80
100
Shift
Log rest length
2.0
2.5
3.0
3.5
4.0
4.5
0
20
40
60
80
100
Shift
Start time
7
8
9
10
11
12
0
20
40
60
80
100
Shift

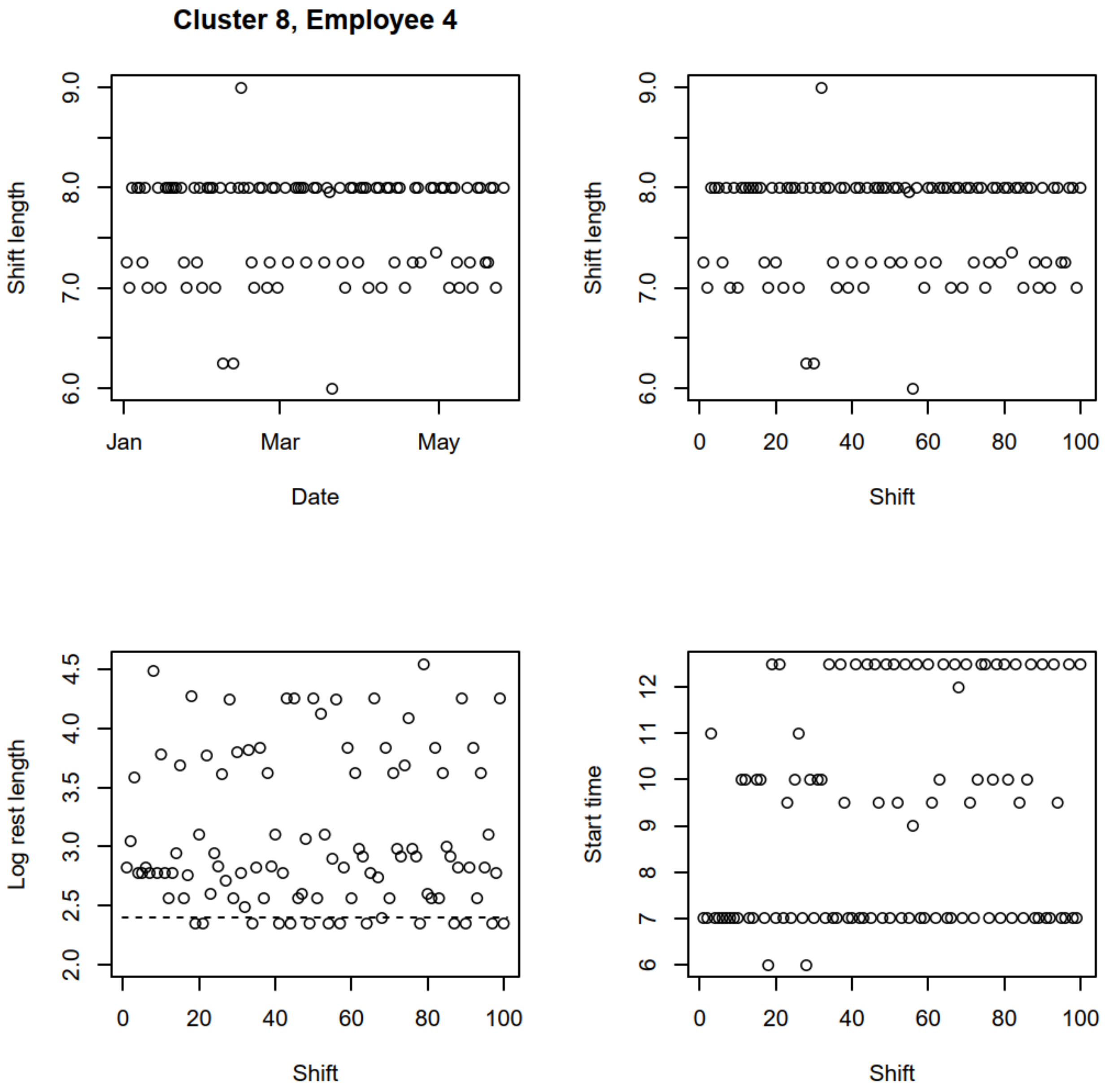
Cluster 8, Employee 4
Shift length
Date
Jan
Mar
May
Shift
Log rest length
Start time
